# Evaluation of confound regression strategies for the mitigation of micromovement artifact in studies of dynamic resting-state functional connectivity and multilayer network modularity

**DOI:** 10.1162/netn_a_00071

**Published:** 2019-02-01

**Authors:** David M. Lydon-Staley, Rastko Ciric, Theodore D. Satterthwaite, Danielle S. Bassett

**Affiliations:** Department of Bioengineering, University of Pennsylvania, Philadelphia, PA, USA; Department of Psychiatry, Perelman School of Medicine, University of Pennsylvania, Philadelphia, PA, USA; Department of Psychiatry, Perelman School of Medicine, University of Pennsylvania, Philadelphia, PA, USA; Department of Bioengineering, University of Pennsylvania, Philadelphia, PA, USA; Department of Electrical and Systems Engineering, University of Pennsylvania, Philadelphia, PA, USA; Department of Neurology, University of Pennsylvania, Philadelphia, PA, USA; Department of Physics and Astronomy, University of Pennsylvania, Philadelphia, PA, USA

**Keywords:** Dynamic functional connectivity, Dynamic networks, Resting-state fMRI, Motion, Artifact, Confound

## Abstract

Dynamic functional connectivity reflects the spatiotemporal organization of spontaneous brain activity in health and disease. Dynamic functional connectivity may be susceptible to artifacts induced by participant motion. This report provides a systematic evaluation of 12 commonly used participant-level confound regression strategies designed to mitigate the effects of micromovements in a sample of 393 youths (ages 8–22 years). Each strategy was evaluated according to a number of benchmarks, including (a) the residual association between participant motion and edge dispersion, (b) distance-dependent effects of motion on edge dispersion, (c) the degree to which functional subnetworks could be identified by multilayer modularity maximization, and (d) measures of module reconfiguration, including node flexibility and node promiscuity. Results indicate variability in the effectiveness of the evaluated pipelines across benchmarks. Methods that included global signal regression were the most consistently effective de-noising strategies.

## Author Summary

Dynamic functional connectivity reflects the spatiotemporal organization of spontaneous brain activity in health and disease, but it can be susceptible to motion artifacts. Here we provide a systematic evaluation of 12 commonly used participant-level confound regression strategies designed to mitigate the effects of micromovements in a sample of 393 youths (ages 8–22 years). Each strategy was evaluated according to the residual association between participant motion and edge dispersion, distance-dependent effects of motion on edge dispersion, the degree to which functional subnetworks could be identified by multilayer modularity maximization, and measures of module reconfiguration, including node flexibility and node promiscuity. Results indicate variability in the effectiveness of the evaluated pipelines across benchmarks. Methods that included global signal regression were the most consistently effective de-noising strategies.

## INTRODUCTION

Resting-state functional magnetic resonance imaging (rs-fMRI) has fundamentally expanded our understanding of the spatiotemporal organization of spontaneous brain activity in health and disease across the life span (Bassett & Bullmore, 2009; Betzel et al., 2014; Biswal, 2012; van den Heuvel & Pol, 2010). To quantify functional connectivity, statistical dependencies (e.g., correlations, coherence; Zalesky, Fornito, & Bullmore, 2012; Z. Zhang, Telesford, Giusti, Lim, & Bassett, 2016) between the blood-oxygen-level-dependent (BOLD) time series of brain regions are typically computed over entire resting-state scans. However, in line with the increasing recognition of the time-varying nature of brain network organization (Calhoun, Miller, Pearlson, & Adali, 2014; Preti, Bolton, & Van De Ville, 2017), rs-fMRI has been increasingly utilized to capture *dynamics* in the organization of spontaneous brain activity over time (Cohen, 2018; Hutchison et al., 2013).

Changes in the connectivity across pairs of nodes (brain regions) are investigated in studies of *dynamic* rs-fMRI, rather than assuming temporal stationarity across entire resting-state scans. This is typically achieved by decomposing the resting-state time series into temporal windows of fixed length. Functional connectivity and dynamic network indices are computed within windows. The gathering of window-specific indices allows an examination of how connectivity changes over time (Allen et al., 2014; Sakoğlu et al., 2010). Emerging network-based tools to describe dynamic rs-fMRI activity (Bassett et al., 2013; Khambhati, Sizemore, Betzel, & Bassett, 2018; Sizemore & Bassett, 2018) have begun to provide important insights into dynamic brain function. These insights include an appreciation for how functional connectivity among brain regions (Allen et al., 2014) and the organization of functional brain network architecture (Chai et al., 2017; Smith et al., 2012) change within persons across the length of scans, how these dynamics relate to normative development (Hutchison & Morton, 2016; Medaglia et al., 2018) and cognition (Bassett, Yang, Wymbs, & Grafton, 2015; Braun et al., 2015; Shine, Koyejo, & Poldrack, 2016), and how they are associated with healthy variations in mood (Betzel, Satterthwaite, Gold, & Bassett, 2017) as well as psychopathology (Braun et al., 2016; Damaraju et al., 2014; Demirtaş et al., 2016).

### The Problem of Motion and Approaches to Mitigating Motion Artifact

Dynamic rs-fMRI approaches reflect the perspective that brain function is quintessentially time-varying. The relatively low demands on participants during acquisition also render dynamic rs-fMRI approaches attractive for use across diverse samples for whom performance on task-based fMRI studies may be difficult. Despite these advantages, dynamic rs-fMRI approaches—like their stationary counterparts—are susceptible to artifacts induced by motion (Laumann et al., 2017). Such artifacts can be mistaken for neural effects (Van Dijk, Sabuncu, & Buckner, 2012) and are particularly problematic in studies of between-person differences, because many between-person differences of interest are correlated with motion (e.g., age, personality, and clinical status; Fair et al., 2013; Satterthwaite, Wolf, et al., 2013; Siegel et al., 2016). Notably, motion artifact is observed in samples free of gross motion, with micromovements as small as 0.1 mm from time point to time point capable of introducing differences in statistics derived from rs-fMRI data (Satterthwaite et al., 2012; Yan et al., 2013).

An important aspect of motion artifact in rs-fMRI is its distance-dependence (Power, Barnes, Snyder, Schlaggar, & Petersen, 2012; Satterthwaite et al., 2012; Van Dijk et al., 2012). Higher levels of motion are associated with greater connectivity in short-range connections and, in some cases, weaker connectivity in long-distance connections. This finding has been of particular concern in the field of developmental cognitive neuroscience wherein a perspective based on early rs-fMRI studies theorized that functional connectivity was modulated in a distance-dependent manner across the life span (e.g., Fair et al., 2009). Since then, it has been demonstrated, through the use of rigorous de-noising procedures, that motion artifact substantially inflated the estimates of age-related, distance-related changes in connectivity (Fair et al., 2013; Satterthwaite, Wolf, et al., 2013).

In response to the problem of in-scanner motion for the study of functional connectivity, there has been a proliferation of techniques geared towards mitigating motion artifacts (Caballero-Gaudes & Reynolds, 2017; Murphy, Birn, & Bandettini, 2013). Confound regression strategies are popular and entail regressing signals thought to be of nonneural origin from the BOLD time series. The residual time series is then used in subsequent analyses. Signals used during confound regression (and often in combination with one another) include realignment parameters, tissue-specific signals, the global signal, signals derived from principal components analysis (PCA), and signals isolated using independent components analysis (ICA).

### Evaluating De-Noising Strategies in Dynamic rs-fMRI

The dizzying array of de-noising strategies provides investigators with many choices during the preprocessing of rs-fMRI data in preparation for dynamic functional connectivity or dynamic network analyses, especially given that the many techniques may be combined within preprocessing pipelines. Recent work has systematically compared the effectiveness of many available techniques in reducing motion-related artifact in static rs-fMRI (Ciric et al., 2017; Parkes, Fulcher, Yucel, & Fornito, 2018). These studies, including work utilizing the same dataset investigated in the present manuscript (Ciric et al., 2017), observed that pipelines with global signal regression (GSR) were among the most effective at minimizing the relationship between connectivity and motion. However, these pipelines revealed a distance-dependent artifact profile. Less effective de-noising pipelines in the context of static functional connectivity included those that used 6 realignment parameter (e.g., Yao et al., 2016), 24 realignment parameter (Friston, Williams, Howard, Frackowiak, & Turner, 1996), and local white matter (WM; Jo, Saad, Simmons, Milbury, & Cox, 2010) confound regression pipelines.

**Figure 1.**
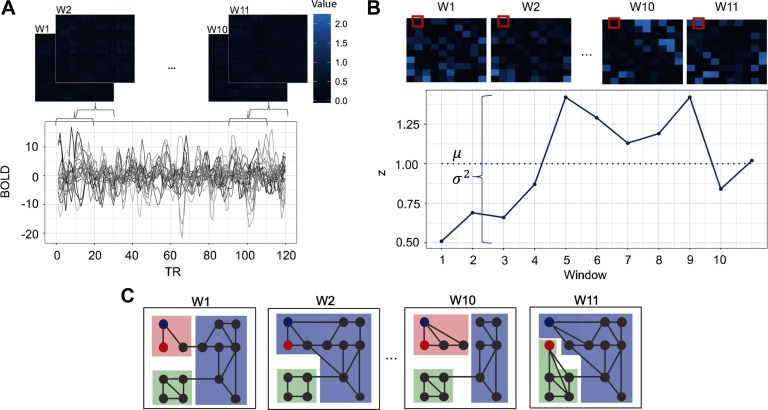
An overview of the construction of time-dependent, whole-brain connectivity matrices and computation of dynamic rs-fMRI indices. Panels (a) through (c) provide an overview of the construction of time-dependent, whole-brain connectivity matrices and computation of dynamic rs-fMRI indices. In panel (a), time courses of blood oxygen level-dependent (BOLD) signals from 270 brain regions were extracted. A sampling of these time courses is plotted (bottom). For each time window (w1, w2, …, w11), node-by-node connectivity matrices were estimated using Pearson correlations, the resulting r values of which were Fisher-z transformed (top). In panel (b), computation of the dispersion index is illustrated. The z value of the edge connecting two nodes at each time window (outlined in red; top) is plotted (continuous blue line; bottom). Dispersion was calculated by dividing the variance () of the 11 z values by the mean () of the 11 z values (mean is indicated by a dotted blue line). Panel (c) illustrates time-dependent community structure over each time window as computed using a multilayer modularity maximization algorithm. Nodes are assigned communities at each time window. A flexible node (blue node) changes community many times (pink, purple, pink, purple). A promiscuous node (red node) also changes communities many times but is marked by allegiances to many communities (pink, purple, pink, green).

The field awaits an extension of this evaluative work of preprocessing strategies to dynamic rs-fMRI. Many dynamic rs-fMRI indices are available. Indices under consideration in the current manuscript (see Figure 1) were chosen based on their use in the existing literature and their observed associations with cognitive processes in previous work. A commonly used measure of dispersion (e.g., Demirtaş et al., 2016), capable of capturing the extent of fluctuations in connectivity between brain regions across time, acted as a dynamic counterpart to static functional connectivity measures that indicate the strength of connectivity between brain regions.

Alongside dispersion, a general measure of dynamic functional connectivity, indices specific to graph or network approaches to understanding the brain were also examined. The focus on network indices reflected the increasing prevalence and utility of conceptualizing the human brain as a complex system composed of individual units (e.g., brain regions) that are connected with one another (Bassett & Sporns, 2017; Bullmore & Sporns, 2009; Calhoun et al., 2014). An additional consideration was the availability of a formal mathematical language and theory to accompany network conceptualizations of the brain (Bullmore & Bassett, 2011; Sporns, 2015).

Within the realm of graph theory,network indices derived from multilayer modularity maximization are of particular interest for probing dynamic functional connectivity. Modularity refers to the ability to decompose the large-scale network of the brain into communities of regions that exhibit dense connectivity with each other and sparse connectivity with regions in other communities. Modularity is a quintessential principle of brain network organization that is thought to confer significant advantages to cognitive functioning (Bassett et al., 2010; Chen, He, Rosa-Neto, Germann, & Evans, 2008; Meunier, Lambiotte, Fornito, Ersche, & Bullmore, 2009; Sporns & Betzel, 2016). The interrogation of modularity in dynamic networks presents challenges that are not encountered in studies of static rs-fMRI. In particular, identifying changes in modules across time requires a way of linking a module at one time point with itself at another time point. Such a linkage is not produced by methods considering single time points separately. Multilayer modularity maximization has emerged as a recent solution to this challenge. In this approach, the network in one time window is linked to networks in adjacent time windows by identity edges that connect a node in one time window to itself in neighboring time windows. Once adjacent time windows are linked, modules are identified (and their temporal variation quantified) by maximizing a multilayer modularity function (Khambhati et al., 2018; Mucha, Richardson, Macon, Porter, & Onnela, 2010). Multilayer modularity approaches to dynamic functional connectivity have, to date, provided insight into a range of cognitive and affective processes, including learning (Bassett et al., 2011), executive functions (Braun et al., 2015), mood (Betzel et al., 2017), and affective disorders (Zheng et al., 2018). Despite the promise of multilayer modularity approaches for providing insight into the spatiotemporal organization of the brain, little work has evaluated the extent to which common preprocessing pipeline choices successfully mitigate motion artifact in the context of these emerging methods.

### The Present Study

To aid in the selection of de-noising pipelines in the study of dynamic rs-fMRI and multilayer network modularity, the present report compared the effectiveness of 12 preprocessing pipelines in the construction of dynamic rs-fMRI indices using a large (*N* = 393) dataset of adolescents and young adults free of gross motion (i.e., with a focus on micromovements). The pipelines evaluated included commonly used techniques, confound regression of varying complexity, PCA- and ICA-based techniques, spatially tailored local white matter regression, and one censoring technique (despiking). GSR was also included in many of the pipelines. Effectiveness was defined by estimates of (a) the residual association between participant motion and edge dispersion, (b) distance-dependent effects of motion on edge dispersion, (c) the degree to which functional subnetworks could be identified by multilayer modularity maximization, and (d) measures of module reconfiguration such as node flexibility and node promiscuity.

## MATERIALS AND METHODS

### Participants and Data Acquisition

The rs-fMRI data were drawn from the Philadelphia Neurodevelopmental Cohort (PNC; Satterthwaite et al., 2016; Satterthwaite et al., 2014) on the basis of age, health, and data quality. Participants provided signed informed consent, with assent and parental consent required for participants under age 18. All procedures were approved by the University of Pennsylvania and the Children’s Hospital of Philadelphia Institutional Review Boards. Selected participants ranged in age from 8 to 22 years, were free from medical conditions that could impact brain function (Merikangas et al., 2010), lacked gross structural brain abnormalities (Gur et al., 2013), were not taking psychotropic medication at the time of the scan, had high-quality imaging data that were free of gross motion (defined as a mean relative root mean squared displacement > 0.2 mm, or > 20 volumes with framewise relative root mean squared displacement > 0.25 mm). Exclusion due to gross motion (*N* = 88; 44 female) allowed an evaluation of the utility of confound regression strategies for minimizing artifact associated with micromovements. Notably, this sample was used in a recent paper that evaluated the effectiveness of confound regression strategies on motion artifact in studies of static functional connectivity (Ciric et al., 2017), facilitating comparisons across static and dynamic connectivity approaches. The sample (59% female) had a mean age of 16.47 (*SD* = 3.55) and a mean relative root mean squared displacement of 0.07 mm (*SD* = 0.40).

Structural and functional data were acquired on a 3T Siemens Tim Trio scanner with a 32-channel head coil (Erlangen, Germany). High-resolution structural images were acquired using a magnetization-prepared, rapid-acquisition gradient-echo (MPRAGE) T1-weighted sequence (*T*_*R*_ = 1,810 ms; *T*_*E*_ = 3.51 ms; FoV = 180 × 240 mm; resolution 1 mm isotropic). Approximately 6 min of resting-state functional data were acquired for each subject using a BOLD-weighted sequence (*T*_*R*_ = 3,000 ms; *T*_*E*_ = 32 ms; FoV = 192 × 192 mm; resolution 3 mm isotropic; 124 spatial volumes). A mock scanning session was conducted using a decommissioned MRI scanner and head coil prior to scanning in order to acclimatize subjects to the MRI environment and to help subjects to learn to remain still during the actual scanning session. During the mock MRI sessions, the MoTrack motion tracking system (Psychology Software Tools, Inc., Sharpsburg, PA) was used to provide feedback to subjects regarding head movement. Prior to data acquisition, subjects’ heads were stabilized in the head coil using one foam pad over each ear and a third pad over the top of the head in order to further minimize motion during the scanning session. During the resting-state scan, a fixation cross was displayed as images were acquired. Subjects were instructed to stay awake, keep their eyes open, fixate on the displayed crosshair, and remain still.

### Structural Image Processing

The “buildTemplateParallelProcedure” in ANTS (Avants, Tustison, Song et al., 2011) was used to generate a study-specific template from a sample of 120 PNC subjects balanced across sex, race, and age bins. Study-specific tissue priors were created using a multi-atlas segmentation procedure (Wang, Cao, & Syeda-Mahmood, 2014). Each subjects’ high-resolution structural image was then processed using the ANTs Cortical Thickness Pipeline (Tustison et al., 2014). After bias field correction (Tustison et al., 2010), each structural image was diffeomorphically registered to the study-specific PNC template using the top-performing SyN deformation (Klein et al., 2009). Study-specific tissue priors were used to guide brain extraction and segmentation of subjects’ structural images (Avants, Tustison, Wu, Cook, & Gee, 2011).

### BOLD Time Series Processing

Resting-state functional images were processed using the XCP Engine (Ciric et al., in press). The XCP Engine was configured to support the pipelines evaluated in this study. Each pipeline was based on de-noising strategies that have previously been described in the neuroimaging literature. Elements of preprocessing common to all pipelines included (a) correction for distortions induced by magnetic field inhomogeneity using FSL’s FUGUE utility, (b) removal of the four initial volumes of each acquisition, (c) realignment of all volumes to a selected reference volume using MCFLIRT (Jenkinson, Bannister, Brady, & Smith, 2002), (d) demeaning and removal of any linear or quadratic trends, (e) coregistration of functional data to the high-resolution structural image using boundary-based registration (Greve & Fischl, 2009), and (f) temporal filtering using a first-order Butterworth filter with a passband between 0.01 and 0.08 Hz, although we note that other frequency ranges may result in more reliable estimations of static network measures (Andellini, Cannatà, Gazzellini, Bernardi, & Napolitano, 2015; Braun et al., 2012). These common processing steps were then followed by the confound regression procedures described below. All regressors were band-pass filtered to retain the same frequency as the data. This was done to prevent frequency-dependent mismatch during confound regression (Hallquist, Hwang, & Luna, 2013). Subject motion was captured using the mean relative RMS (root-mean-squared; Jenkinson et al., 2002) displacement as calculated during time series realignment using MCFLIRT (Satterthwaite, Elliott, et al., 2013; Satterthwaite et al., 2012).

### Overview of Confound Regression Strategies

The pipelines evaluated in the present manuscript variably incorporated realignment parameters, tissue-specific signals, GSR, PCA, ICA, and censoring approaches to motion mitigation. We provide a brief overview of these approaches before specifying the evaluated pipelines.

#### Realignment parameters.

During the rigid body realignment of functional images to correct for head movement (via the use of MCFLIRT in the present manuscript), 6 realignment parameters (3 translations and 3 rotations) are produced. These 6 realignment parameters are commonly used as nuisance regressors in dynamic rs-fMRI preprocessing (e.g., Yao et al., 2016). The first temporal derivatives of the 6 realignment parameters are also often included to account for time lags in the effects of motion (e.g., Vergara, Mayer, Damaraju, & Calhoun, 2017). Other expansions on realignment parameters include creating quadratic terms for the 12 parameters (the 6 original realignment parameters and their first temporal derivatives), yielding 24 parameters in total (Friston et al., 1996).

#### Tissue-specific signals.

Tissue-specific signals are also used as nuisance regressors in dynamic rs-fMRI studies and include time series from white matter and cerebrospinal fluid (e.g., Di & Biswal, 2015). The inclusion of mean WM and CSF signals is intended to reduce the impact of nonneural BOLD fluctuations that may be attributed to motion, scanner artifacts, and physiological signals that are typically not of interest (e.g., respiration; Windischberger et al., 2002). Use of local WM regressors is also possible and entails using the average signal within an eroded WM mask within a 15 mm radius of each gray matter voxel (Jo et al., 2010).

#### Global signal regression.

The global signal is the time series of signal intensity averaged across all voxels in the brain. The global signal is used in dynamic rs-fMRI studies as a nuisance regressor (e.g., Liu & Duyn, 2013) as it is strongly linked to nonneuronal processes that include head motion, respiratory patterns, and hardware artifacts (Power, Plitt, Laumann, & Martin, 2017). Use of GSR has been a source of debate in the rs-fMRI literature (Murphy & Fox, 2016), with concerns that it may remove signals of interest (Schölvinck, Maier, Frank, Duyn, & Leopold, 2010) and bias group comparisons (Gotts et al., 2013; Saad et al., 2012). Recent studies comparing a number of de-noising pipelines on motion artifact in rs-fMRI indicate that the use of GSR in preprocessing pipelines is effective at reducing the association between motion and functional connectivity but may have some undesirable effects on the distance-dependent effects of motion (Ciric et al., 2017; Parkes et al., 2018).

#### Principal component analysis.

PCA approaches have been used in the preprocessing of dynamic rs-fMRI (e.g., Kucyi & Davis, 2014). The aim in PCA approaches is to isolate regions of the image that are strongly driven by motion and other sources of nonneural signal, derive principal components from these noise regions, and include them as nuisance regressors. Two common variants of this approach—anatomic COMPCOR (aCOMPCOR) and temporal COMPCOR (tCOMPCOR)—isolate noise regions of interest by either (a) performing PCA on voxelwise CSF and eroded WM signals, or (b) identifying high-noise regions by their temporal standard deviation, respectively (Behzadi, Restom, Liau, & Liu, 2007; Cheng et al., 2017). PCA has been observed to be more effective than tissue-mean signal regression approaches at removing motion artifact from rs-fMRI data (Muschelli et al., 2014).

#### Independent component analysis.

ICA has been used in dynamic rs-fMRI to identify distinct functional networks that differ based on their temporal independence (Smith et al., 2012). As well as being used as a technique to identify functional networks at the group level, ICA is used in the preprocessing of dynamic rs-fMRI to identify noise time series that are then regressed from the data (Barber, Lindquist, DeRosse, & Karlsgodt, 2018). ICA decomposes the rs-fMRI data into multiple components, some of which will be components of interest (i.e., those reflecting BOLD signal) and some of which will reflect artifactual processes. Once decomposed, identification of artifactual components is required. Two common approaches to noise component identification are ICA-FIX (Salimi-Khorshidi et al., 2014) and ICA-AROMA (Pruim et al., 2015). ICA-FIX requires manual labeling of the components derived from ICA in a training dataset, whereas ICA-AROMA uses a predefined set of features that are automatically extracted from the image to identify noise. Once the data are decomposed and noise components are identified, the noise components are regressed from the rs-fMRI data.

#### Censoring methods.

Beyond confound regression techniques, temporal censoring methods are available to reduce the impact of motion. In censoring approaches, motion-corrupted volumes are identified and either removed from the data or interpolated. Despiking identifies outliers on a voxelwise basis based on the intensity of each voxel’s time series, and interpolates over the outliers. Scrubbing and spike regression, in contrast, censor complete volumes based on deviation from an *a priori* motion threshold (Power et al., 2012; Satterthwaite, Elliott, et al., 2013). While despiking has been used in studies of dynamic rs-fMRI (e.g., Allen et al., 2014; Damaraju et al., 2014), more extreme censoring methods, including scrubbing, are often explicitly avoided because of concerns over interrupting the temporal autocorrelation structure of the data (e.g., H. Zhang, Chen, Zhang, & Shen, 2017) and the potential issue of resulting in sliding windows of differing lengths (Hutchison et al., 2013).

### Pipelines Used in the Present Study

**Figure 2.**
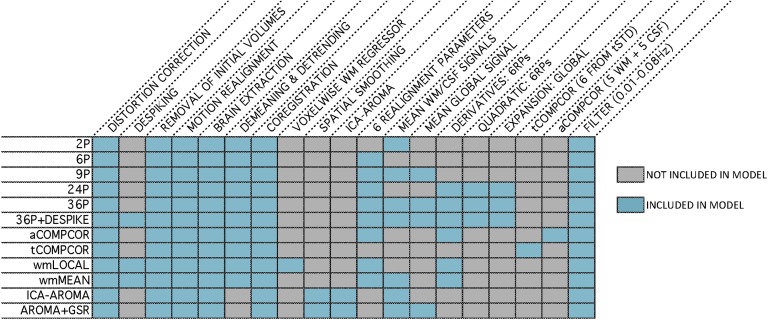
Schematic of the 12 de-noising pipelines evaluated in the present study. For each of the 12 pipelines (left), the table details the included processing procedures and confound regressors (top).

Twelve commonly used de-noising pipelines were selected for evaluation (Figure 2; see also Ciric et al., 2017, for more information).

***Pipeline 1.*** Pipeline 1 (2P) functioned as a base pipeline for comparison with more complex confound regression pipelines. It employed two physiological time series: mean signal in white matter and mean signal in cerebrospinal fluid.
***Pipeline 2.*** Pipeline 2 (6P) used six motion estimates derived from MCFLIRT realignment as regressors.
***Pipeline 3.*** Pipeline 3 (9P) combined the two physiological time series from Pipeline 1 and the six motion estimates from Pipeline 2 with GSR.
***Pipeline 4.*** Pipeline 4 (24P) was an expansion of Pipeline 2 that included six temporal derivatives, six quadratic terms, and six quadratic expansions of the derivatives of the six motion estimates, resulting in a total of 24 regressors.
***Pipeline 5.*** Pipeline 5 (36P) was an expansion of Pipeline 3, incorporating derivatives, quadratic terms, and squares of derivatives of six motion, two physiological time series, and GSR.
***Pipeline 6.*** Pipeline 6 (36P+despike) included the 36 regressors of Pipeline 5 in addition to despiking (Cox, 1996).
***Pipeline 7.*** Pipeline 7 (aCOMPCOR) used five principal components each from the WM and CSF, in addition to motion estimates and their temporal derivatives.
***Pipeline 8.*** Pipeline 8 (tCOMPCOR) used six principal components from high-variance voxels.
***Pipeline 9.*** Pipeline 9 (wmLOCAL) used a voxelwise, localized WM regressor in addition to motion estimates, their temporal derivatives, and despiking.
***Pipeline 10.*** Pipeline 10 (wmMEAN) used the mean signal across the WM instead of the voxelwise, localized WM regressor of Pipeline 9. It also included motion estimates, their temporal derivatives, and despiking.
***Pipeline 11.*** Pipeline 11 (ICA-AROMA) used an ICA-based procedure for removal of motion-related variance from BOLD data, alongside mean WM and CSF regressors.
***Pipeline 12.*** Pipeline 12 (AROMA+GSR) combined ICA-AROMA with GSR.

### Data Analysis

Each de-noising pipeline was evaluated according to the following benchmarks: (a) the residual association between participant motion and edge dispersion, (b) distance-dependent effects of motion on edge dispersion, (c) the degree to which functional subnetworks could be identified by multilayer-modularity maximization, and (d) measures of module reconfiguration such as node flexibility and node promiscuity. In this section, we provide details on dynamic network creation before providing an overview of each benchmark.

#### Dynamic network creation.

Brain networks for each de-noising pipeline were defined with a commonly used, whole-brain, spherical node parcellation (Power et al., 2011). For each network, the mean time series for each node was calculated from the de-noised residual data. The time series was divided into *T* = 11 sliding time windows, each 20 TRs (60 s) in duration, with 50% overlap. The choice of window length was consistent with work indicating that the majority of dynamic functional connectivity work to date has employed 30- to 60-s windows, with most studies using 20 data points per window (Preti et al., 2017). The use of the upper bound of 60 s and 20 TRs was chosen because evidence suggests that window lengths of 60 s or greater may be required to balance the capacity to capture dynamics in BOLD signal and the ability to obtain accurate connectivity estimates within windows (Leonardi & Van De Ville, 2015), although we acknowledge that shorter windows can also be useful for some questions (Braun et al., 2016; Braun et al., 2015). An added benefit of this choice was that it also allowed us to achieve even window lengths across the experiment. Within each window, edges between all nodes were estimated via Pearson correlation. Negative correlations were set to 0, similar to previous functional connectivity studies (e.g., Chan, Park, Savalia, Petersen, & Wig, 2014; Grady, Sarraf, Saverino, & Campbell, 2016), to eliminate potential misinterpretation of negative edge weights (see Supporting Information, Network Density Across Pipelines, for information on the number of negative edges, network density, and average positive edge weight of networks across pipelines; Lydon-Staley, Ciric, Satterthwaite, & Bassett, 2019). In additional analyses, negative correlations were retained (see Supporting Information, Results with Negative Edge Weights Included; Lydon-Staley et al., 2019). A Fisher *z*-transformation was then performed on all correlations. The result was a time-ordered set of functional connectivity matrices for each subject and de-noising pipeline that was used in the creation of dynamic functional connectivity and network indices (see Figure 1A).

#### Edge dispersion measure.

The extent of fluctuations in connectivity between individual edges across time was assessed using a dispersion index (e.g., Demirtaş et al., 2016). For each de-noising pipeline, the mean and the variance of the functional connectivity of each edge across all sliding windows were computed for each subject. This allowed the creation of a dispersion index

$$C_{i}=\frac{\sigma_{i}^{2}}{\mu_{i}},$$

where *C*_*i*_ is the dispersion of edge *i* based on the variance of edge *i* divided by the mean value of edge *i* (see Figure 1B). Dispersion values of edges that showed no fluctuations across windows were set to 0.

#### Edge dispersion outcome: Dispersion-motion associations.

For each edge, we computed the correlation coefficient between the dispersion of that edge and subject motion. To control for the potential influence of demographic factors, partial correlations that accounted for subject age and sex were calculated. This procedure resulted in a distribution of dispersion-motion correlations for each pipeline. From these distributions, two outcomes of pipeline efficacy in minimizing motion artifact were computed: (a) the *number of edges significantly related to motion*, computed after controlling for multiple comparisons via the false discovery rate (FDR at *α* = 0.05; Benjamini & Hochberg, 1995); (b) the median absolute value of all *dispersion-motion* correlations.

#### Edge dispersion outcome: Distance-dependent motion effects.

Motion artifact has been demonstrated to be associated with the distance between nodes (Power et al., 2012; Satterthwaite et al., 2012). Short-distance edges exhibit stronger connectivity while long-distance edges exhibit weaker connectivity as a result of subject motion. The center of mass of each node was used to obtain a distance matrix *D* where element *D*_*ij*_ indicated the Euclidean distance between the centers of mass of nodes *i* and *j*. Correlations between the distance separating each pair of nodes and the dispersion-motion correlation of the edge connecting these nodes was then estimated to provide an outcome measure of the extent to which each de-noising pipeline mitigated *distance-dependent* effects of motion. Statistical comparisons of the distance-dependent motion effects of the pipelines were achieved using tests of the equality of correlation coefficients following Steiger (1980) and using the *cocor* package in *R* (Diedenhofen & Musch, 2015).

#### Network modularity measure.

The degree to which there were structured subnetworks in the connectivity matrices from each pipeline was characterized using a common dynamic community detection technique to maximize multilayer modularity. We first transform the ordered set of adjacency matrices (windows 1–11 in the present case) into a multilayer network (Bassett et al., 2013; Mucha et al., 2010). In this multilayer network, the graph in one time window is linked to the graph in adjacent time windows via identity edges that connect a node in one time window to the same node in neighboring time windows. Multilayer modularity is defined as

$$Q=\frac{1}{2\mu }\sum_{ijlr}\left\{(A_{ijl}-\gamma_{l}P_{ijl})\delta_{lr}+\delta_{ij}\omega_{jlr}\right\}\delta (g_{il},g_{jr}),$$

where *A*_*ijl*_ is the edge weight between nodes *i* and node *j* in time window *l*; *P*_*ijl*_ is the expected weight of the edge connecting node *i* and node *j* under a specified null model; *γ*_*l*_ is a structural resolution parameter of layer *l* that tunes the number of communities identified; *g*_*il*_ is the community assignment of node *i* in layer *l*; *g*_*jr*_ gives the community assignment of node *j* in layer *r*; *δ*(*g*_*il*_, *g*_*jr*_) = 1 if *g*_*il*_ = *g*_*jr*_ and 0 otherwise; the total network edge weight is *μ* = $\frac{1}{2}\sum_{jr}\kappa_{jr}$; the strength of node *j* in layer *l* is *κ*_*jl*_ = *κ*_*jl*_ + *c*_*jl*_; the intralayer strength of node *j* in layer *l* is *κ*_*jl*_ = $\sum_{i}A_{ijl}$; and the interlayer strength of node *j* in layer *l* is *c*_*jl*_ = $\sum_{r}\omega_{jlr}$.

Multilayer modularity maximization was implemented in MATLAB (Jeub, Bazzi, Jutla, & Mucha, 2011) and was applied to each subject’s functional connectivity matrices separately for each de-noising pipeline. The algorithm was applied with a default structural resolution parameter, *γ*_*l*_, of 1 and an interlayer strength parameter, *ω*_*jlr*_, of 1. As the heuristic is nondeterministic, the algorithm was iterated 100 times for each subject for each de-noising pipeline. This procedure resulted in 100 *Q* values for each subject for each de-noising pipeline as well as 100 *n* × *m* matrices, where *n* is the number of nodes (264) and *m* is the number of sliding windows (i.e., 11), indicating the community allegiance of each node during each sliding window.

#### Network modularity outcome: Subnetwork identification.

The mean *Q* value across the 100 iterations was taken as an outcome measure of *how separable the brain networks were into subnetworks* after de-noising.

#### Network modularity outcome: *Q*-motion associations.

Partial correlations between *Q* and participant motion were calculated while controlling for participant age and sex in order to provide an indication of the extent to which each de-noising pipeline minimized the *association between motion and modularity*.

#### Node flexibility and promiscuity measures.

The matrices indicating community assignment of nodes across sliding windows were taken forward to create additional indices related to node flexibility and node promiscuity (see Figure 1C). Node flexibility captures the number of times a node changes communities across time, normalized by the number of times the node could have changed communities (Bassett et al., 2011). Formally, node flexibility is defined as

$$f_{i}=\frac{m}{T-1},$$

where *f*_*i*_ is the flexibility of node *i*, *m* is the number of times node *i* changed communities, and *T* is the number of sliding windows. Using the Network Community Toolbox (http://commdetect.weebly.com), node flexibility was calculated for each subject across each pipeline as the average flexibility value across the 100 iterations of the dynamic community detection procedure. Flexibility *F* of the dynamic network as a whole was calculated as the average of *f*_*i*_ over all nodes.

Node promiscuity captures additional information concerning the dynamics of network nodes and is defined as

$$\psi_{i}=\frac{k}{K},$$

where *ψ*_*i*_ is the promiscuity of node *i*, *k* is the number of communities in which node *i* participates, and *K* is the total number of communities (Papadopoulos, Puckett, Daniels, & Bassett, 2016). A node with high promiscuity exhibits allegiances to many communities across sliding windows. Using the Network Community Toolbox (http://commdetect.weebly.com), node promiscuity was calculated for each subject across each pipeline as the average promiscuity value across the 100 iterations of the dynamic community detection procedure. Promiscuity Ψ of the dynamic network as a whole was calculated as the average of *ψ*_*i*_ over all nodes.

#### Node flexibility and promiscuity outcome: Flexibility-motion and promiscuity-motion associations.

For each node, a correlation was computed between the flexibility of that node and participant motion. To control for the potential influence of demographic factors, partial correlations that accounted for participant age and sex were calculated. This resulted in a distribution of flexibility-motion correlations for each pipeline. From these distributions, two measures of pipeline efficacy in minimizing motion artifact were computed: (a) the *number of nodes significantly related to motion*, computed after controlling for multiple comparisons via the false discovery rate (FDR; Benjamini & Hochberg, 1995); (b) the median absolute value of all *flexibility-motion* correlations. Further, partial correlations between *F* and participant motion were calculated while controlling for participant age and sex in order to provide an indication of the extent to which each de-noising pipeline minimized the association between *motion and global flexibility*.

The analyses conducted for flexibility were repeated for promiscuity. This resulted in a distribution of promiscuity-motion correlations for each pipeline as well as a measure of the *number of nodes significantly related to motion* and the median absolute value of all *flexibility-motion* correlations. The association between *motion and global promiscuity* was also computed.

## RESULTS

In this section we present the performance of the de-noising pipelines for mitigating motion artifact on (a) the residual association between participant motion and edge dispersion, (b) distance-dependent effects of motion on edge dispersion, (c) the degree to which functional subnetworks could be identified by multilayer-modularity maximization, and (d) measures of module reconfiguration such as node flexibility and node promiscuity.

### Edge Dispersion–Motion Association Is Minimal Across Pipelines

**Figure 3.**
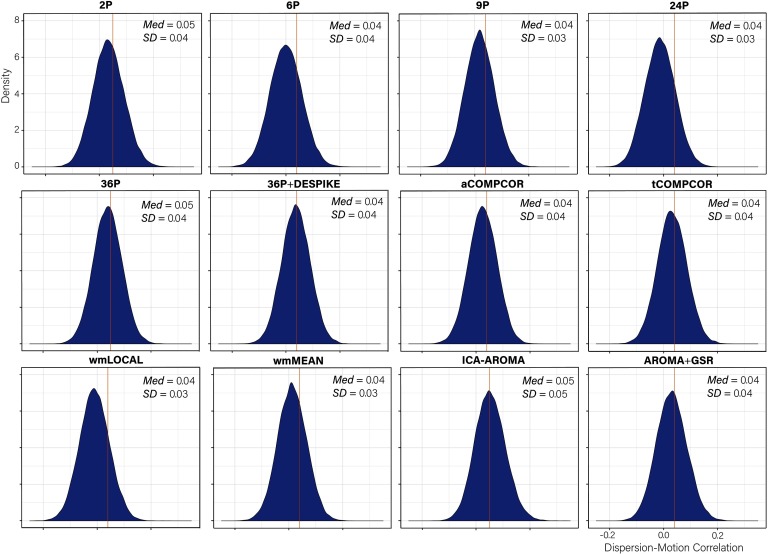
Distributions of all edgewise dispersion-motion correlations after de-noising for each de-noising pipeline. The median absolute value of the correlation and the standard deviation of the correlation are both displayed in the top right of each panel.

Distributions of *dispersion-motion* correlations are presented in Figure 3. Paired-sample *t* tests indicated significant differences in the mean *dispersion-motion* correlation across all pipelines (Table S6; see Lydon-Staley et al., 2019). The median absolute *dispersion-motion* correlations ranged between 0.04 and 0.05, indicating small associations between *dispersion* and participant motion following the application of the de-noising pipelines.

**Figure 4.**
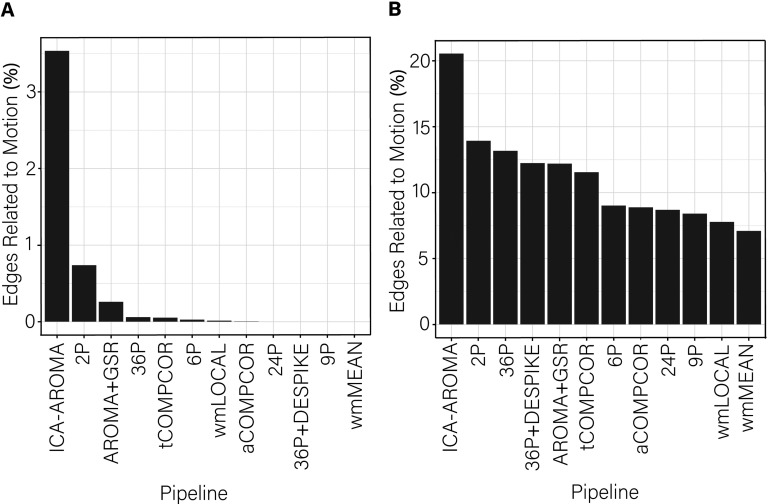
Percentage of edges significantly related to motion after de-noising for the dispersion index. More effective de-noising pipelines reduced the relationship between dispersion and motion. Bars are ordered such that the least effective de-noising pipelines are on the left and the most effective are on the right. Panel A illustrates the results after correcting for multiple comparisons. Panel B depicts the results without controlling for multiple comparisons.

While the range of *dispersion-motion* correlations was small across pipelines, there was some heterogeneity in the performance of the various pipelines. This heterogeneity was most notable in the percentage of edges whose dispersion was related to motion (Figure 4A). The percentage of edges whose dispersion was associated with motion was generally small (i.e., <1%). However, the ICA-AROMA pipeline emerged as a clear outlier with over 3% of edges having dispersion that remained associated with subject motion following de-noising. Minor differences among the best performing pipeline emerged when the false discovery rate to control for multiple comparisons was applied (Figure 4A) versus when it was not applied (Figure 4B).

### Dispersion Distance-Dependent Motion Artifact Varies Across Pipelines

**Figure 5.**
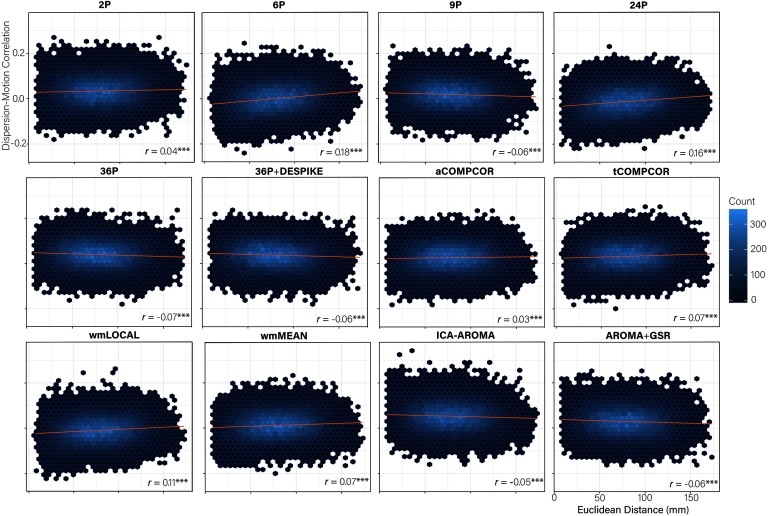
Hexbin plots of the association between the Euclidean distance separating nodes (mm) and the magnitude of the dispersion-motion correlation between the edge connecting the nodes (y-axis). A trend line for each pipeline is indicated in blue and the magnitude of the correlation is presented in the bottom right of each panel. *Note*: ***p < 0.001.

More marked differences emerged in the performance of the 12 pipelines for the extent to which *distance-dependent effects of motion* on dispersion were present following de-noising (Figure 5). Correlations between the Euclidean distance separating the nodes and the magnitude of the dispersion-motion correlation for the edge connecting those nodes ranged between −0.07 and 0.18. Tests of equality of correlations revealed significant differences in the *distance-dependent effects of motion* across many pipelines (Table S7; Lydon-Staley et al., 2019). Little evidence for nonequivalent correlations emerged among the 9P, 36P, and 36P+DESPIKE, AROMA+GSR, ICA-AROMA pipelines (*r* values between −0.07 and −0.05) and the tCOMPCOR and wmMEAN pipelines (*r* values both equal to 0.07). Particularly poorly performing pipelines included the 6P, 24P, and wmLOCAL pipelines.

### Marked Heterogeneity in Subnetwork Identification Across Pipelines

**Figure 6.**
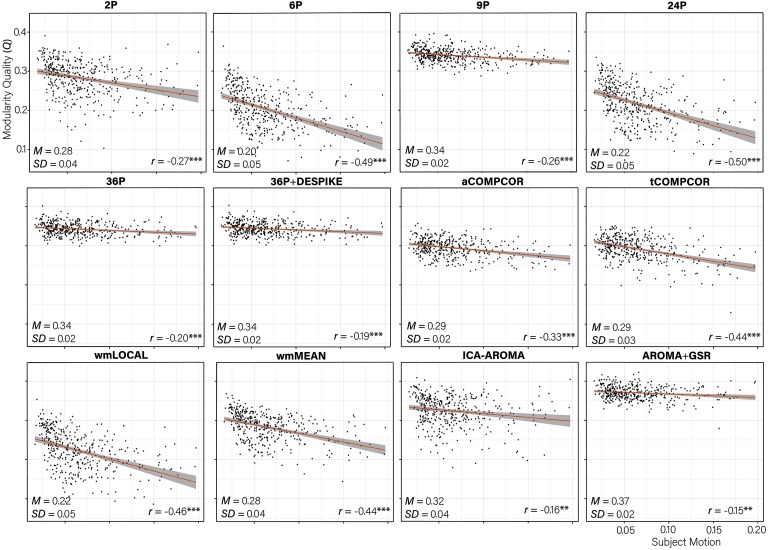
Scatterplot of the association between subject motion (x-axis) and modularity quality (*Q*, y-axis) with trend line. The partial correlation between subject motion and *Q* controlling for age and sex is presented in the bottom right of each panel. The mean and standard deviation of the *Q* value for each pipeline is presented in the bottom left of each panel. ***p < 0.001; **p < 0.01.

There were marked differences across de-noising pipelines in the extent to which subnetworks were identifiable, as operationalized by the *network modularity quality (Q)*. Paired sample *t* tests revealed significant differences across the *Q* values of most pipelines (Table S8; Lydon-Staley et al., 2019). The difference between the *Q* values from the 2P and the wmMEAN pipeline was not significant. The 6P pipeline was the least effective at allowing the identification of subnetworks, exhibiting a mean *Q* value of 0.20 (see Figure 6). AROMA+GSR emerged as the most effective pipeline, with a mean *Q* value 0.37. Notably, of the top five pipelines, four made use of GSR. Supplementary analyses (see Supporting Information, Mean *Q* Across Pipelines Controlling for Graph Density; Lydon-Staley et al., 2019) examined the effect of pipeline on *Q* controlling for age, sex, and network density, and indicated that differences in *Q* values across pipelines were not simply a product of differences in network density.

### Negative *Q*-Motion Associations Reduced by Effective Pipelines

Correlations between *subject motion and Q* ranged between −0.50 and −0.15, with greater subject motion associated with lower *Q* values in all instances (Figure 6). Pipelines that were the least effective at allowing the identification of subnetworks tended to be the pipelines that were the least effective at mitigating subject motion artifacts, *r*(10) = 0.89, *p* < 0.001. For example, pipelines 6P and 24P performed poorly across both indices. In contrast, AROMA+GSR and 36P+DESPIKE performed consistently well.

### Node Flexibility–Motion Correlations Generally Small Across Pipelines

**Figure 7.**
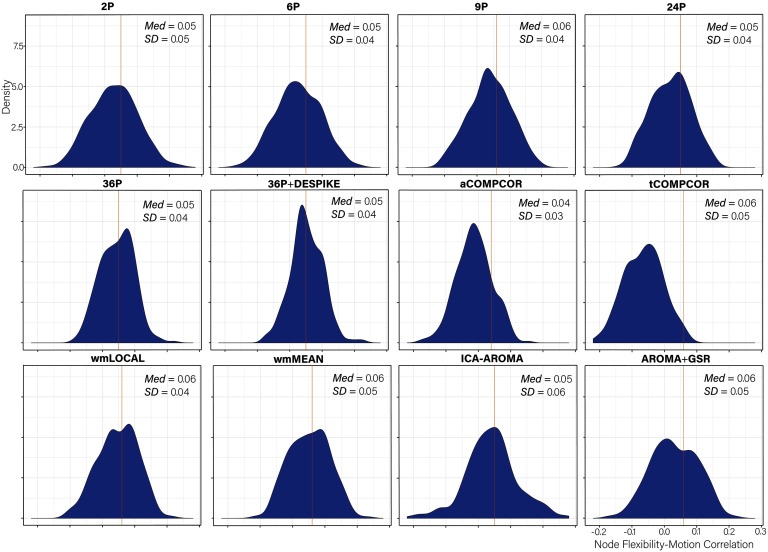
Distributions of all node flexibility-motion correlations after de-noising for each pipeline. The median absolute value of the correlation and the standard deviation of the correlation is displayed in the top right of each panel. Panels are ordered by the magnitude of the absolute correlation.

Distributions of *node flexibility–motion* correlations for each pipeline are presented in Figure 7. Paired sample *t* tests comparing the mean node flexibility–motion correlations across pipelines are presented in Table S9 (Lydon-Staley et al., 2019). Absolute median correlations were small in magnitude across pipelines and ranged between 0.04 and 0.06.

**Figure 8.**
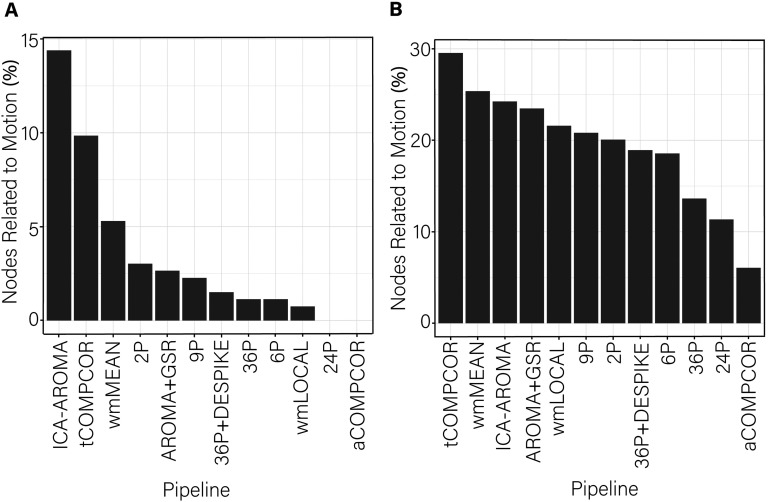
Percentage of nodes significantly related to motion after de-noising for node flexibility. More effective de-noising pipelines reduced the relationship between flexibility and motion. Bars are ordered such that the least effective de-noising pipelines are on the left and the most effective are on the right. Panel A illustrates the results after correcting for multiple comparisons. Panel B depicts the results without controlling for multiple comparisons.

**Figure 9.**
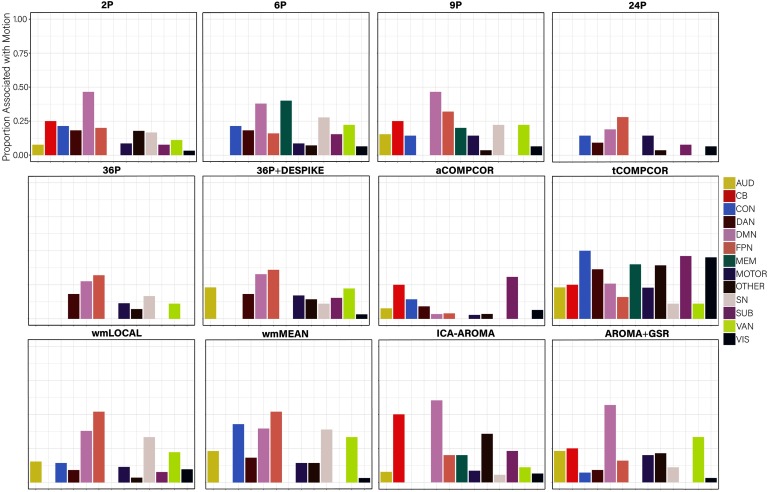
The proportion of nodes within each subnetwork of the Power et al. (2011) parcellation with flexibility values significantly related to subject motion, without controlling for multiple comparisons. AUD = auditory; CB = cerebellum; CON = cingulate-opercular network; DAN = dorsal attention network; DMN = default mode network; FPN = frontoparietal network; MEM = memory network; SN = salience; SUB = subcortical; VAN = ventral attention network; VIS = visual network.

Examining the *percentage of nodes related to subject motion* following de-noising (Figure 8), there was greater variability across pipelines in the extent to which subject motion artifacts were reduced. ICA-AROMA and tCOMPCOR emerged as the least successful pipelines, with over 8% of nodes displaying flexibility values that were significantly associated with motion after de-noising. No nodes displayed flexibility values that were significantly associated with subject motion for the 24P and aCOMPCOR pipelines. Minor differences among the best performing pipeline emerged when the false discovery rate to control for multiple comparisons was applied (Figure 8A) versus when it was not applied (Figure 8B). The proportion of nodes within each subnetwork of the Power et al. (2011) parcellation significantly associated with subject motion are presented in Figure 9 to provide an indication of which subnetworks were most contaminated by residual motion.

**Figure 10.**
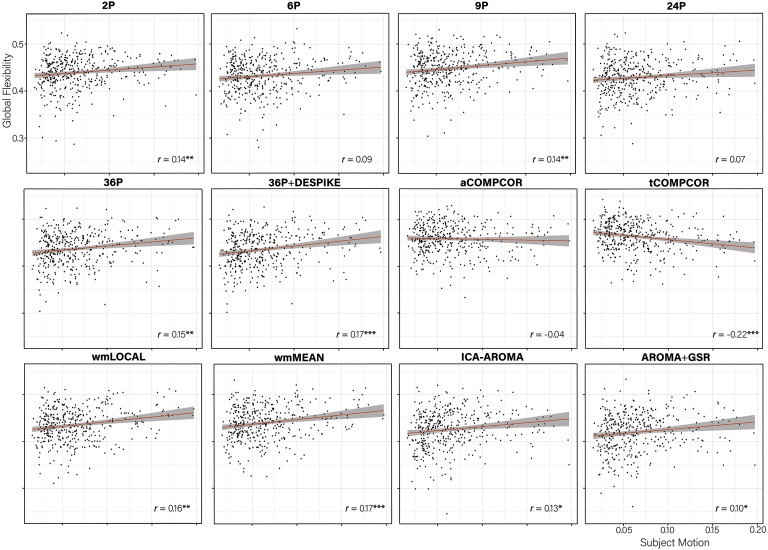
Scatterplot of the association between subject motion (x-axis) and global flexibility (y-axis) with trend line. The partial correlation between subject motion and global flexibility controlling for age and sex is presented in the bottom right of each panel. ***p < 0.001; **p < 0.01; *p < 0.05.

When examining the association between *global flexibility and subject motion* (Figure 10), no significant associations between subject movement and global flexibility emerged for three (6P, 24P, aCOMPCOR) of the 12 pipelines; tCOMPCOR emerged as the least effective pipeline for mitigating the association between subject motion and global flexibility.

### Node Promiscuity–Motion Correlations Generally Small Across Pipelines

**Figure 11.**
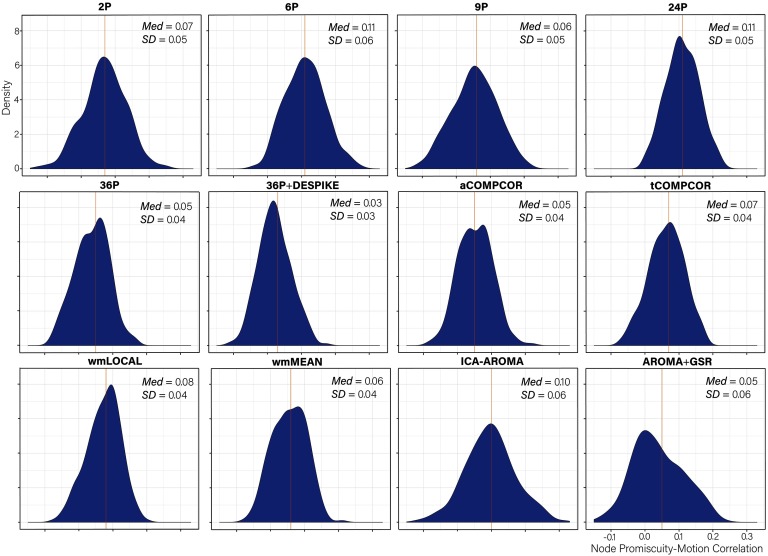
Distributions of all node promiscuity-motion correlations after de-noising for each pipeline. The median absolute value and the standard deviation of the correlation are both displayed in the top right of each panel.

Distributions of *node promiscuity–motion* correlations are presented in Figure 11. Paired sample *t* tests comparing the mean node promiscuity–motion correlations across pipelines are presented in Table S10 (Lydon-Staley et al., 2019). Absolute median correlations were larger in magnitude than those observed for node flexibility, but were generally small. The values across pipelines ranged between 0.03 and 0.11 with the 36P+DESPIKE exhibiting the best performance, and with both the 6P and 24P pipelines exhibiting the worst performance.

**Figure 12.**
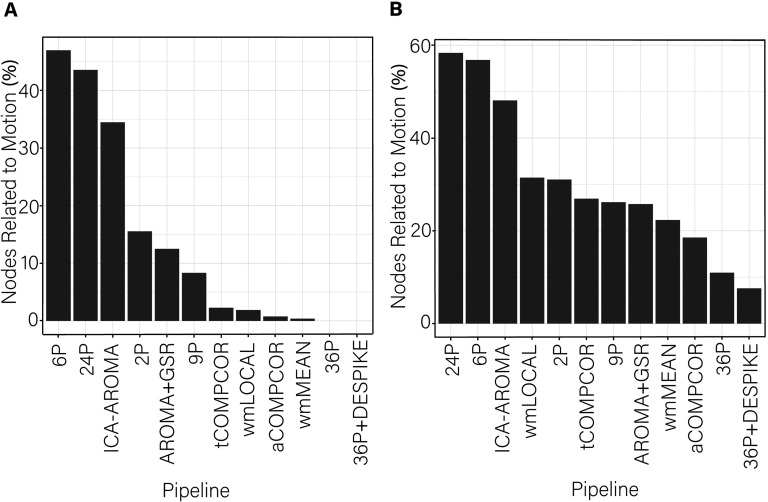
Percentage of nodes significantly related to motion after de-noising for node promiscuity. More effective de-noising pipelines reduced the relationship between promiscuity and motion. Bars are ordered such that the least effective de-noising pipelines are on the left and the most effective are on the right. Panel A illustrates the results after correcting for multiple comparisons. Panel B depicts the results without controlling for multiple comparisons.

**Figure 13.**
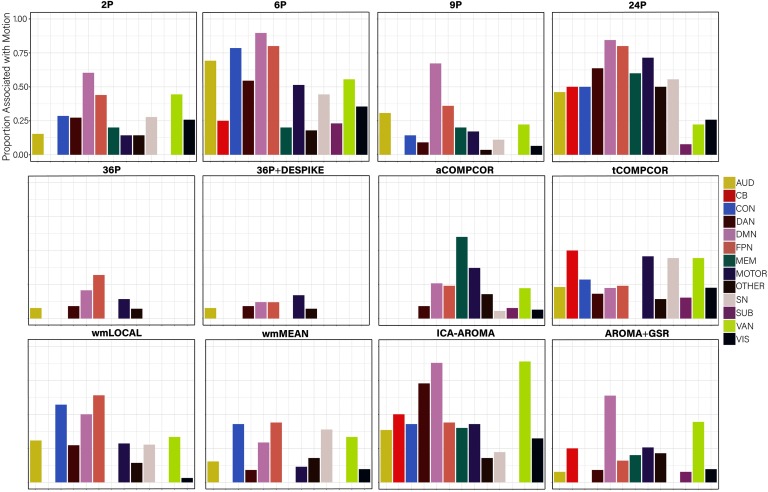
The proportion of nodes within each subnetwork of the Power et al. (2011) parcellation with promiscuity values significantly related to subject motion, without controlling for multiple comparisons. AUD = auditory; CB = cerebellum; CON = cingulate-opercular network; DAN = dorsal attention network; DMN = default mode network; FPN = frontoparietal network; MEM = memory network; SN = salience; SUB = subcortical; VAN = ventral attention network; VIS = visual network.

Examining the percentage of nodes significantly associated with subject motion following de-noising, there was marked variability in the effectiveness of reducing motion artifact across pipelines (Figure 12). Similar to findings for flexibility, virtually no nodes displayed promiscuity values that were significantly associated with motion after use of the aCOMPCOR pipeline. No nodes were associated with motion in the 36P and 36P+DESPIKE pipelines. Also similar was the poor performance of the ICA-AROMA pipeline. The 24P pipeline exhibited the least effectiveness at reducing the association between subject motion and node promiscuity, exhibiting the highest percentage of nodes associated with subject motion. The 6P pipeline also performed poorly. Minor differences among the best performing pipeline emerged when the false discovery rate to control for multiple comparisons was applied (Figure 12A) versus when it was not applied (Figure 12B). The proportion of nodes within each subnetwork of the Power et al. (2011) parcellation significantly associated with subject motion are presented in Figure 13.

**Figure 14.**
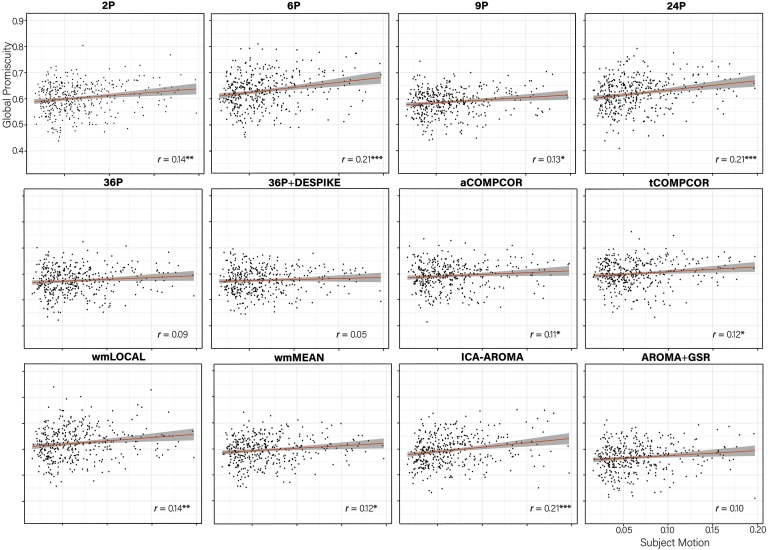
Scatterplot of the association between subject motion (x-axis) and global promiscuity (x-axis) with trend line. The partial correlation between subject motion and global promiscuity controlling for age and sex is presented in the bottom right of each panel. ***p < 0.001; **p < 0.01; *p < 0.05.

When examining the association between *global promiscuity and subject motion* (Figure 14), pipelines exhibiting worse performance in terms of the number of nodes significantly associated with subject motion (i.e., ICA-AROMA, 6P, and 24P) also exhibited the largest correlations among subject motion and global promiscuity. Three of the four pipelines using GSR (36P, 36P+DESPIKE, AROMA+GSR) showed no significant correlations with motion.

## DISCUSSION

The capacity for in-scanner participant motion to contaminate rs-fMRI data has been increasingly appreciated, with many de-noising strategies emerging in response (Power, Schlaggar, & Petersen, 2015; Satterthwaite et al., 2017). To aid in the selection of de-noising pipelines in the study of dynamic rs-fMRI data, the present study evaluated the performance of 12 de-noising pipelines in mitigating motion artifact associated with micromovements across a range of dynamic (mainly multilayer) functional connectivity indices. Results indicated heterogeneity in pipeline performance according to the following benchmarks: (a) the residual association between participant motion and edge dispersion, (b) distance-dependent effects of motion on edge dispersion, (c) the degree to which functional subnetworks could be identified by multilayer modularity maximization, and (d) measures of module reconfiguration such as node flexibility and node promiscuity. Results also indicated that the effectiveness of the de-noising pipelines evaluated differed across dynamic functional connectivity indices. Results, implications, and study limitations are discussed below.

Dispersion has been used in dynamic functional connectivity studies to capture the extent to which the coupling between regions of interest fluctuates at rest (e.g., Demirtaş et al., 2016). When examining the percentage of edges significantly related to motion, ICA-AROMA was the least successful at mitigating motion, performing worse than the baseline 2P pipeline. However, ICA-AROMA was among the most successful pipelines in terms of removing distance-dependent motion artifact, performing equivalently to the 9P, 36P, 36P+ DESPIKE, and AROMA+GSR pipelines. Notably, the dispersion-motion correlations were much lower in the current dynamic functional connectivity case than has been observed for equivalent edge-based indices of functional connectivity that consider the entire rs-fMRI data (e.g., Ciric et al., 2017). Given that the dispersion-motion correlation magnitudes and the percentage of edges associated with motion were generally low, one may wish to prioritize the ability for pipelines to mitigate distance-dependent motion artifacts in dispersion indices. The most consistently effective pipelines for mitigating the problem of motion for dispersion across the three performance benchmarks were pipelines including GSR and aCOMPCOR.

While the majority of de-noising pipelines were effective at dealing with motion for edge dispersion, striking differences in the performance of de-noising pipelines emerged in the extent to which subnetworks could be identified in the processed data. In terms of modularity quality (*Q*), pipelines incorporating GSR (e.g., AROMA+GSR, 36P, 36P+DESPIKE) resulted in the highest *Q* across the board. Notable also was the reduced heterogeneity across participants in terms of *Q* values that resulted from pipelines incorporating GSR, with *Q* values for these pipelines exhibiting less variability around the mean relative to pipelines without GSR (Figure 6). Pipelines including GSR were also generally effective at mitigating participant motion–*Q* associations, with AROMA+GSR exhibiting the lowest correlations. The least effective pipelines included 6P and 24P pipelines, which exhibited the greatest modularity-motion correlations. White matter, tissue-based regression pipelines also performed poorly. Findings are generally in accordance with de-noising pipeline performance on modularity indices in static functional connectivity (Ciric et al., 2017).

Node-level dynamics were captured using flexibility and promiscuity indices (Bassett et al., 2011; Papadopoulos et al., 2016). For node flexibility, ICA-AROMA, tCOMPCOR, and wmMEAN pipelines exhibited the greatest percentage of nodes associated with motion and also exhibited the highest global flexibility–subject motion correlations. ICA-AROMA also performed poorly for promiscuity, as did the 6P and 24P pipelines. Although 24P and 6P performed relatively well in terms of outcomes associated with flexibility, we caution that flexibility and promiscuity are somewhat dependent on the ability for pipelines to effectively identify subnetworks given that both flexibility and promiscuity indices measure the participation of nodes in communities identified via multilayer modularity. As such, when considering flexibility and promiscuity, pipelines that perform consistently well in subnetwork detection as well as flexibility and promiscuity (e.g., 36P, 36P+DESPIKE) are recommended.

Taken together, the 12 de-noising pipelines evaluated here showed varying effectiveness across their capacity to mitigate the effect of motion and to maximize the ability to identify subnetworks in dynamic rs-fMRI. ICA-AROMA emerged as an interesting case. ICA-AROMA has performed relatively well in previous evaluations in the context of static connectivity (Ciric et al., 2017; Parkes et al., 2018), and it also performed well on a number of benchmarks in the current study (e.g., performing equivalently to pipelines using GSR in reducing distance-dependent dispersion-motion artifact). For flexibility and promiscuity indices, however, ICA-AROMA performed poorly, indicating that pipelines may not perform equally well across static and dynamic functional connectivity contexts.

Investigators may wish to tailor their de-noising approach to the dynamic functional connectivity index of most interest to them. Alternatively, de-noising approaches that show less effective performance across a range of indices (e.g., 6P, 24P, tCOMPCOR, wmLOCAL) may be avoided in favor of pipelines exhibiting more effective performance across the many different indices evaluated (e.g., 36P+DESPIKE, aCOMPCOR). We caution that, although approaches using GSR performed relatively well in the current case, there is evidence that static graph indices resulting from pipelines incorporating GSR may be less reliable (as assessed using test-retest) than those without GSR (see Andellini et al., 2015, for review). It will be important for future work to examine the extent to which limitations in reliability associated with GSR generalize from static to dynamic functional connectivity.

### Limitations

The findings of the present report should be evaluated in light of study limitations. The choice of indices to identify effective de-noising procedures were intuitive and complemented those used in static rs-fMRI benchmarking approaches (Ciric et al., 2017). However, the lack of a ground truth remains a challenge in the evaluation of pipeline effectiveness. Although the present report included many de-noising strategies, it is not an exhaustive evaluation of all available artifact-control strategies. ICA-FIX, for example, was not evaluated. However, as it requires manual labeling and training data, it is a less common method of de-noising relative to the strategies examined in the present report. In constructing indices of dynamic functional connectivity, a sliding window approach using Pearson correlations was taken. This is a common approach, but alternative approaches exist to which the current results may not generalize. These alternatives include the use of wavelet-based methods to clean the time series prior to estimating functional connectivity (Z. Zhang et al., 2016), other measures of functional connectivity such as coherence (Bassett et al., 2011), and approaches that do not rely on sliding windows to capture changes in functional connectivity (e.g., dynamic connectivity regression; Cribben, Haraldsdottir, Atlas, Wager, & Lindquist, 2012). Examining motion artifact in a sample free of gross motion allowed a focus on confound regression strategies for the mitigation of motion artifact introduced through micromovements. The results here may not generalize to samples with more extensive motion.

The dynamic functional connectivity indices examined in the present manuscript are commonly used, especially edge dispersion (e.g., Demirtaş et al., 2016). The focus on graph indices (e.g., modularity, flexibility) reflects increasing interest and utility of network indices to describe brain function and recent findings indicating their importance for understanding cognitive processes (Bassett et al., 2011; Muldoon & Bassett, 2016; Telesford et al., 2017). Dynamic network indices at smaller topological scales (e.g., temporal clustering), larger topological scales (e.g., small-worldness), and measures that may combine the two (e.g., temporal small-worldness; Sizemore & Bassett, 2018) were beyond the scope of the current manuscript and will require evaluation in future work. Finally, while the examination of dynamic connectivity has provided insight into the spatiotemporal organization of spontaneous brain activity, we direct readers to important considerations about the potential physiological underpinnings of dynamic rs-fMRI (Laumann et al., 2017; Liégeois, Laumann, Snyder, Zhou, & Yeo, 2017).

### Conclusion

In sum, the present study highlights the varying effectiveness of commonly used de-noising pipelines in studies of dynamic functional connectivity and dynamic network architecture in rs-fMRI. By evaluating many pipelines across common indices used in dynamic functional connectivity and network neuroscience studies, the present report provides investigators with a means to evaluate the relative strengths and weaknesses of available de-noising pipelines.

## ACKNOWLEDGMENTS

The content is solely the responsibility of the authors and does not necessarily represent the official views of any of the funding agencies.

## AUTHOR CONTRIBUTIONS

David Martin Lydon-Staley: Formal analysis; Investigation; Methodology; Writing – original draft; Writing – review & editing. Rastko Ciric: Data curation; Formal analysis; Writing – review & editing. Theodore D. Satterthwaite: Conceptualization; Data curation; Funding acquisition; Supervision; Writing – original draft; Writing – review & editing. Danielle S. Bassett: Conceptualization; Funding acquisition; Investigation; Methodology; Resources; Supervision; Writing – original draft; Writing – review & editing.

## FUNDING INFORMATION

Danielle S. Bassett, John D. and Catherine T. MacArthur Foundation (http://dx.doi.org/10.13039/100000870). Danielle S. Bassett, Alfred P. Sloan Foundation (http://dx.doi.org/10.13039/100000879). Danielle S. Bassett, ISI Foundation. Danielle S. Bassett, Paul Allen Foundation. Danielle S. Bassett, Army Research Laboratory (http://dx.doi.org/10.13039/100006754), Award ID: W911NF-10-2-0022. Danielle S. Bassett, Army Research Laboratory (http://dx.doi.org/10.13039/100006754), Award ID: W911NF-14-1-0679. Danielle S. Bassett, Army Research Office (http://dx.doi.org/10.13039/100000183), Award ID: W911NF-16-1-0474. Danielle S. Bassett, Army Research Office (http://dx.doi.org/10.13039/100000183), Award ID: W911NF-17-2-0181. Danielle S. Bassett, Office of Naval Research (http://dx.doi.org/10.13039/100000006). Danielle S. Bassett, National Institute of Mental Health (http://dx.doi.org/10.13039/100000025), Award ID: 2-R01-DC-009209-11. Danielle S. Bassett, National Institute of Mental Health (http://dx.doi.org/10.13039/100000025), Award ID: R01-MH112847. Danielle S. Bassett, National Institute of Mental Health (http://dx.doi.org/10.13039/100000025), Award ID: R01-MH107235. Danielle S. Bassett, National Institute of Mental Health (http://dx.doi.org/10.13039/100000025), Award ID: R21-M-MH-106799. Danielle S. Bassett, National Institute of Child Health and Human Development (http://dx.doi.org/10.13039/100000071), Award ID: 1R01HD086888-01. Danielle S. Bassett, National Institute of Neurological Disorders and Stroke (http://dx.doi.org/10.13039/100000065), Award ID: R01-NS099348. Danielle S. Bassett, National Science Foundation (http://dx.doi.org/10.13039/100000001), Award ID: BCS-1441502. Danielle S. Bassett, National Science Foundation (http://dx.doi.org/10.13039/100000001), Award ID: BCS-1430087. Danielle S. Bassett, National Science Foundation (http://dx.doi.org/10.13039/100000001), Award ID: PHY-1554488. Danielle S. Bassett, National Science Foundation (http://dx.doi.org/10.13039/100000001), Award ID: BCS-1631550. Theodore D. Satterthwaite, National Institute of Mental Health (http://dx.doi.org/10.13039/100000025), Award ID: R01MH107703. Theodore D. Satterthwaite, National Institute of Mental Health (http://dx.doi.org/10.13039/100000025), Award ID: R21MH106799. Theodore D. Satterthwaite, National Institute of Mental Health (http://dx.doi.org/10.13039/100000025), Award ID: R01MH112847. Theodore D. Satterthwaite, Lifespan Brain Institute at Penn/CHOP.

## TECHNICAL TERMS

Functional connectivity: Statistical dependence between activity levels of brain regions.
Dynamic functional connectivity: Changes in the statistical dependence between activity levels of brain regions across time.
Layer: An element of multilayer networks. In this paper, layers represent graphs in time windows of 60 s.
Multilayer brain network: A brain network with more than one layer. Layers are connected to one another via interlayer edges that link nodes across different layers.
Edge dispersion: Edge dispersion captures the extent of fluctuations in the functional connectivity of an edge across time. It is computed by dividing the variance of an edge by the mean value of the edge.
Node flexibility: Node flexibility captures the number of times a node changes communities across time, normalized by the number of times the node could have changed communities.
Node promiscuity: Node promiscuity captures the number of communities in which a node participates across time. A node with high promiscuity participates in many communities across time.
