# Supplementary material for: Evaluation of confound regression strategies for the mitigation of micromovement artifact in studies of dynamic resting-state functional connectivity and multilayer network modularity
Source: Netw Neurosci. 2019 Feb 1;3(2):427–54. doi: 10.1162/netn_a_00071 (PMC6370491; doi:10.1162/netn_a_00071)
Supplement: Supplementary file 1 [file netn-03-427-s001.pdf]

Lydon, D. M., Ciric, R., Satterthwaite, T.D., & Bassett, D.S. (2019). Supporting Information for "Evaluation of Confound Regression Strategies for the Mitigation of Micromovement Artifact in Studies of Dynamic Resting State Functional Connectivity and Multilayer Network Modularity." *Network Neuroscience* 3(2), 427–454.
https://doi.org/10.1162/netn_a_00071

SUPPLEMENTAL MATERIAL

Network Density Across Pipelines

The proportion of negative edges across pipelines is presented in Supplemental Figure 1. The proportion of negative edges out of the full 766,656 (264 x 264 nodes x 11 sliding windows) was computed for each subject for each pipeline. In line with previous observations (e.g., Murphy et al., 2009), pipelines making use of GSR (e.g., 9P, 36P) tended to have a greater proportion of negative edges relative to pipelines that did not use GSR (e.g., 2P).

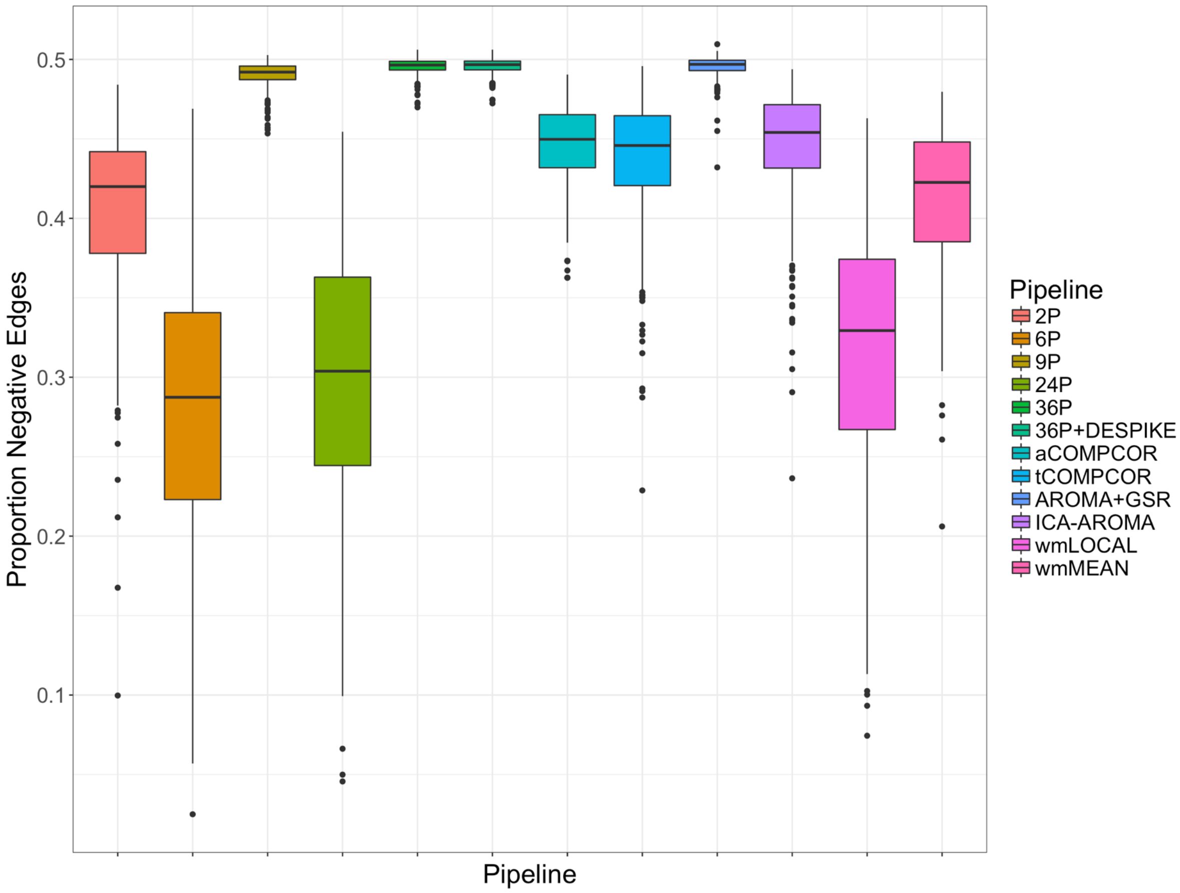

Figure S1. Proportion negative edges across all pipelines.

Network density across pipelines is presented in Supplemental Figure 2. The proportion of non-zero, positive edges out of the full 766,656 (264 x 264 nodes x 11 sliding windows) was computed for each subject for each pipeline. As expected based on the number of negative edges across pipelines, pipelines making use of GSR (e.g.,9P) tended to have lower density networks relative to pipelines that did not use GSR (e.g., 2P).

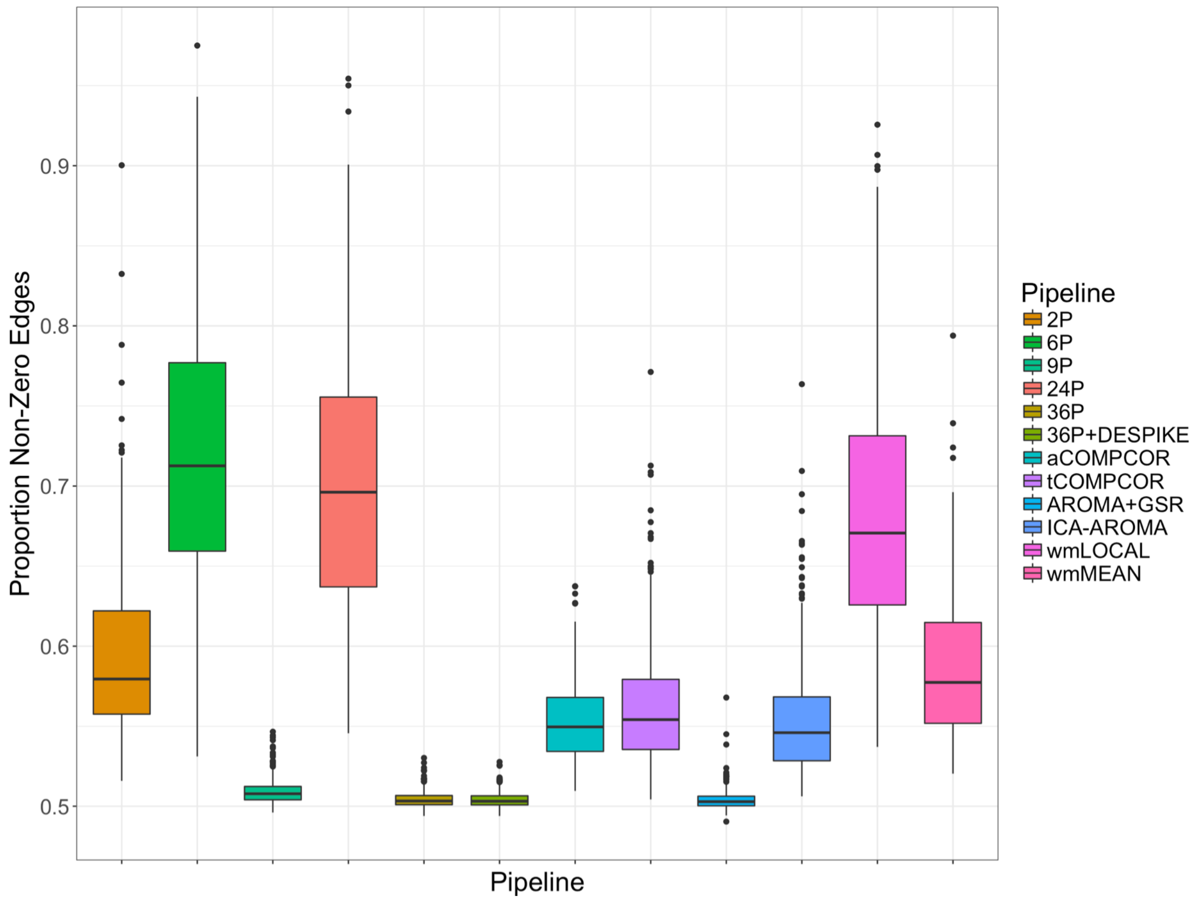

Figure S2. Proportion non-zero edges across all pipelines.

The average positive edge weight of networks across pipelines is presented in Supplemental Figure 3. The average of all non-zero, positive edges was computed for each subject for each pipeline.

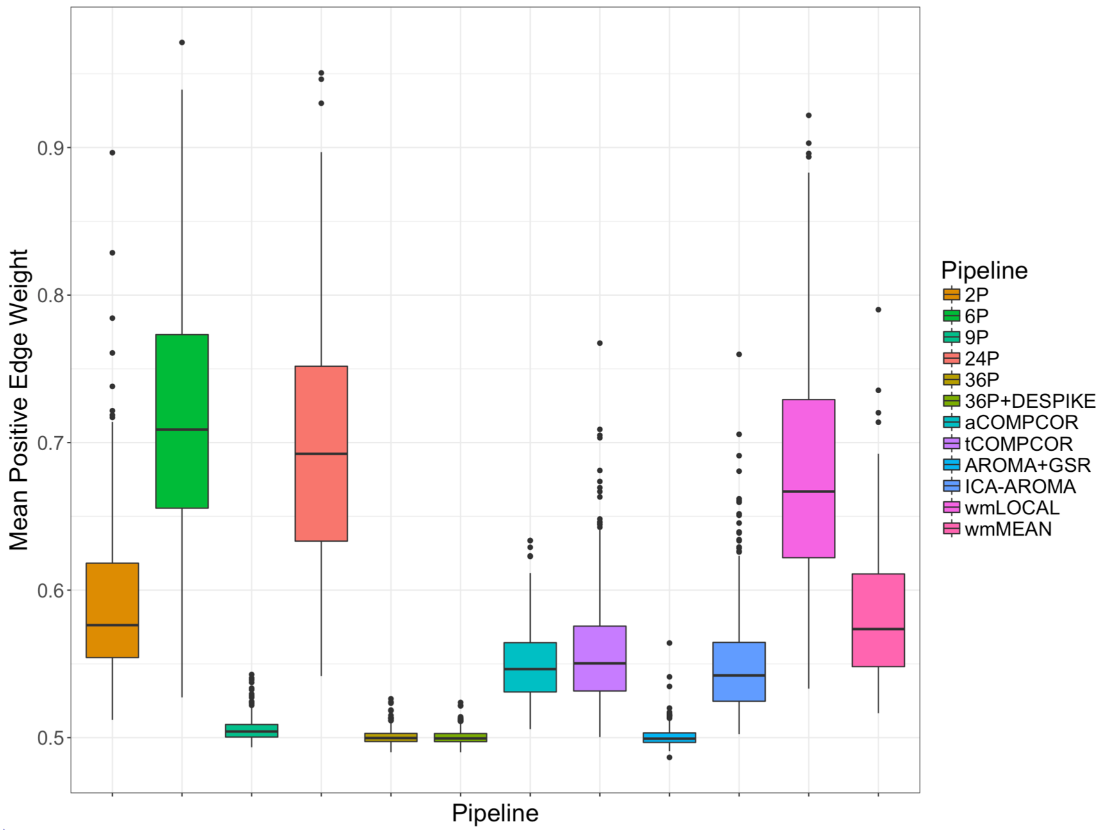

Figure S3. Mean positive edge weight across all pipelines.

Results with Negative Edge Weights Included

This section reports results parallel to those in the manuscript using functional connectivity estimates that retained negative edge weights. The dispersion measure used in the main manuscript was not appropriate for use with data including negative edge weights as a negative ratio of variance to the mean would be uninterpretable. As such, the dispersion measures used in the case of networks including negative edge weights was the standard deviation of the edge weights.

Edge Dispersion-Motion Association is Minimal Across Pipelines

Distributions of *dispersion-motion* correlations are presented in Figure S4. Paired-sample *t*-tests indicated significant differences in the mean *dispersion-motion* correlation across all pipelines (Table S1). The median absolute *dispersion-motion* correlations ranged between 0.04 and 0.10, indicating small associations between *dispersion* and participant motion following the application of the de-noising pipelines.

Table S1

Paired samples *t*-test comparisons of the mean dispersion-motion correlations across all pipelines

	2P	6P	9P	24P	36P	36PDESPIKE	aCOMPCOR	tCOMPCOR	wmLOCAL	wmMEAN	ICAAROMA
**2P**	-										
**6P**	-28.02	-									
**9P**	144.89	186.99	-								
**24P**	65.60	108.07	-57.44	-							
**36P**	45.79	73.92	-77.02	-21.19	-						
**36PDESPIKE**	55.65	84.65	-66.20	-9.44	30.06	-					
**aCOMPCOR**	82.77	116.83	-39.45	19.96	40.07	28.82	-				
**tCOMPCOR**	41.70	73.28	-99.04	-32.52	-12.46	-22.93	-54.04	-			
**wmLOCAL**	119.59	183.10	-7.04	58.87	69.76	58.82	32.85	85.52	-		
**wmMEAN**	145.05	195.73	21.18	81.45	95.84	84.56	58.68	110.25	41.12	-	
**ICAAROMA**	-94.10	-66.06	-215.64	-153.85	-131.31	-141.39	-168.33	-127.01	-203.69	-228.20	-
**AROMAGSR**	36.13	59.56	-98.46	-35.03	-15.97	-26.21	-53.20	-4.06	-83.60	-110.114	134.16

Notes: Elements of matrix represent *t*-values. Degrees of freedom for all *t*-tests = 34715. Columns represent reference level (i.e., positive t-values indicate higher mean dispersion-motion correlations for the pipeline indicated by the column versus the row heading). All *p*-values were <0.001.

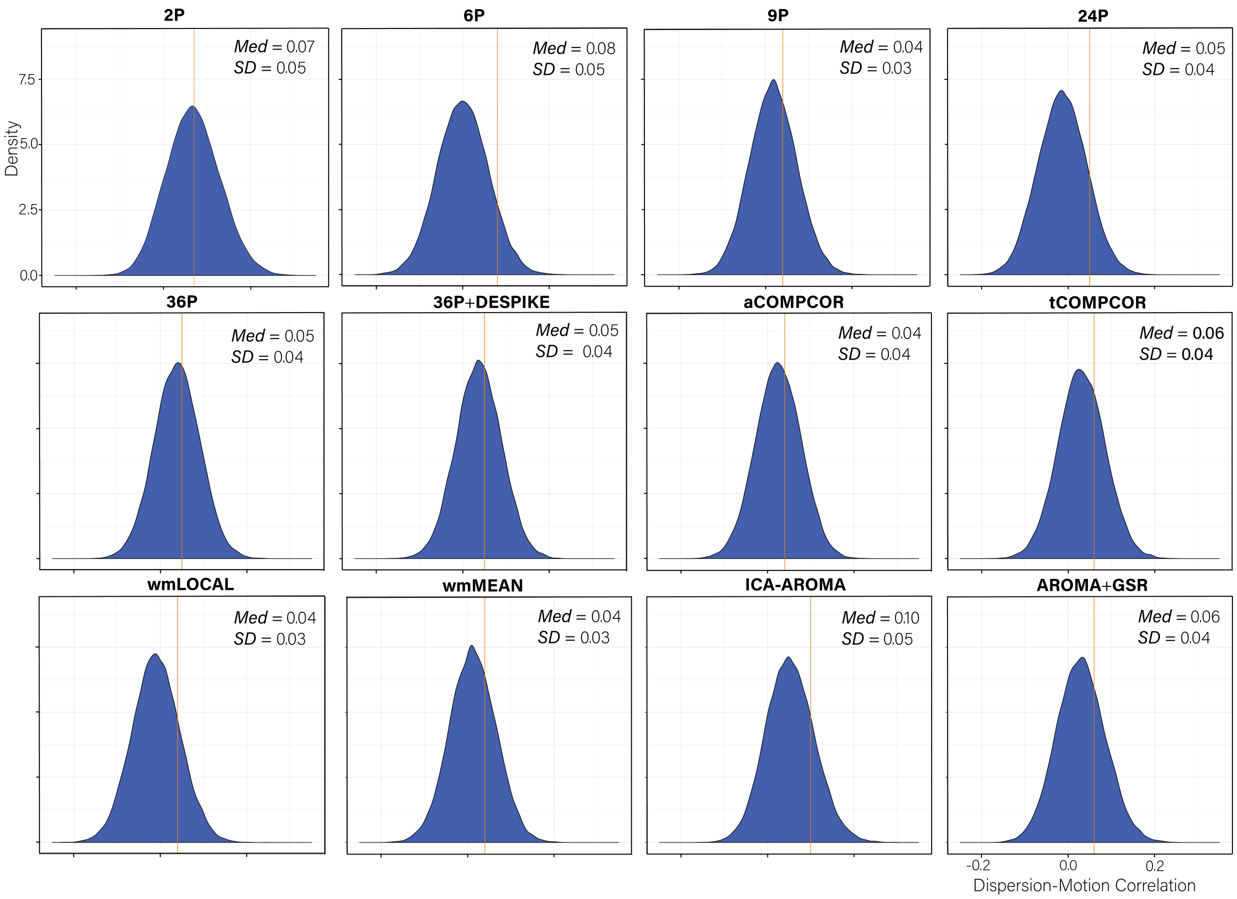

Figure S4. Distributions of all edgewise dispersion-motion correlations after de-noising for each de-noising pipeline. The median absolute value of the correlation and the standard deviation of the correlation is displayed in the top right of each panel.

While the range of *dispersion-motion* correlations was small across pipelines, heterogeneity in pipeline performance was most notable in the percent of edges whose dispersion was related to motion (Figure S5). The percent of edges whose dispersion was associated with motion was generally small (i.e., < 1%). However, the ICA-AROMA pipeline emerged as a clear outlier with over 30% of edges having dispersion values that remained associated with subject motion following de-noising. The performance of ICA-AROMA in the case in which negative edge weights were retained, paralleled its performance in the case in which negative edge weights were not retained (Figure 4 in main manuscript).

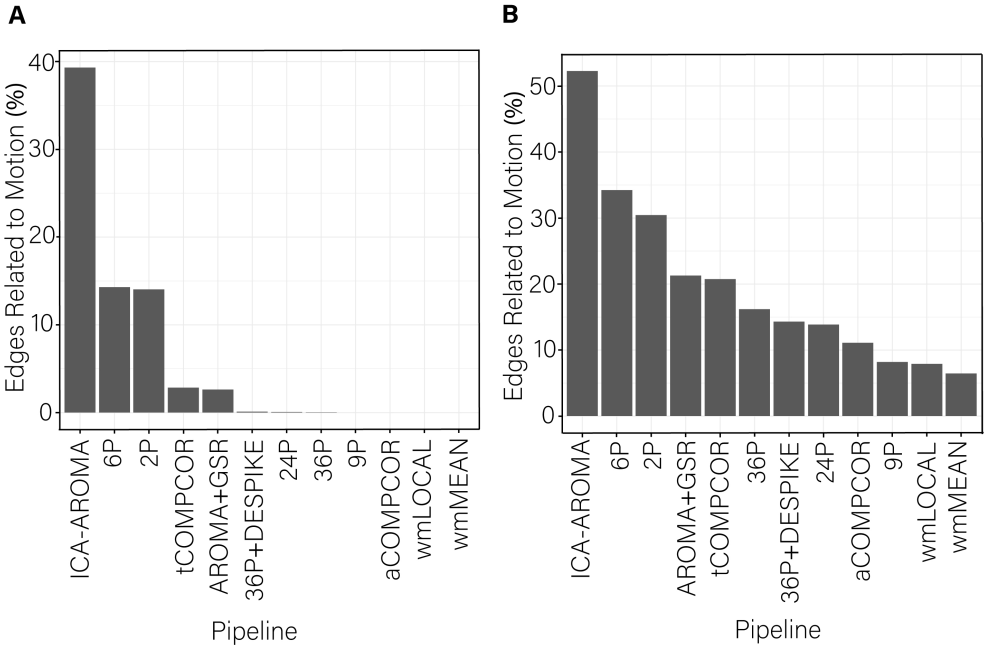

Figure S5. Percent of edges significantly related to motion after de-noising for the dispersion index. More effective de-noising pipelines reduced the relationship between dispersion and motion. Bars are ordered such that the least effective de-noising pipelines are on the left and the most effective are on the right. Panel A illustrates the results after correcting for multiple comparisons. Panel B depicts the results without controlling for multiple comparisons.

Dispersion Distance-Dependent Motion Artifact Varies Across Pipelines

Correlations between the Euclidean distance separating the nodes and the magnitude of the dispersion-motion correlation for the edge connecting those nodes ranged between -0.09 and 0.02 (Figure S6). Tests of equality of correlations revealed significant differences in the *distance-dependent effects of motion* across many pipelines (Table S2). Particularly poorly-performing pipelines included the 6P, 24P, wmLOCAL, and tCOMPCOR pipelines. These pipelines were also poorly-performing pipelines in the context in which negative edge weights were not retained (Figure 5 in main manuscript).

Table S2

Tests of equality of correlation coefficients representing the association between the Euclidean distance separating nodes (mm) and the magnitude of the *dispersion-motion* correlation between the edges connecting the nodes.

	2P	6P	9P	24P	36P	36PDESPIKE	aCOMPCOR	tCOMPCOR	wmLOCAL	wmMEAN	ICAAROMA
**2P**	-										
**6P**	13.50	-									
**9P**	9.37	-4.19	-								
**24P**	12.21	1.73	5.02	-							
**36P**	7.66	-2.57	0.66	-4.41	-						
**36PDESPIKE**	6.24	-4.06	-0.90	-5.99	-4.09	-					
**aCOMPCOR**	9.22	-1.47	2.01	-3.08	1.23	2.78	-				
**tCOMPCOR**	17.37	4.56	8.29	2.32	6.29	7.79	5.60	-			
**wmLOCAL**	14.53	3.51	6.84	1.46	5.34	6.85	4.45	-1.11	-		
**wmMEAN**	3.61	-8.69	-4.90	-9.82	-4.79	-3.25	-6.34	-11.94	-17.10	-	
**ICAAROMA**	7.05	-4.71	-1.31	-5.91	-1.78	-0.36	-2.97	-8.60	-7.22	2.83	-
**AROMAGSR**	2.04	-9.27	-6.22	-10.20	-5.96	-4.52	-7.29	-13.49	-11.55	-1.56	-4.87

Notes: *n* = 34716. Elements of matrix represent *z*-values. Columns represent reference level (i.e., positive t-values indicate higher distance-motion correlations for the pipeline indicated by the column versus the row heading). Shaded rows indicate non-significant differences between pipelines.

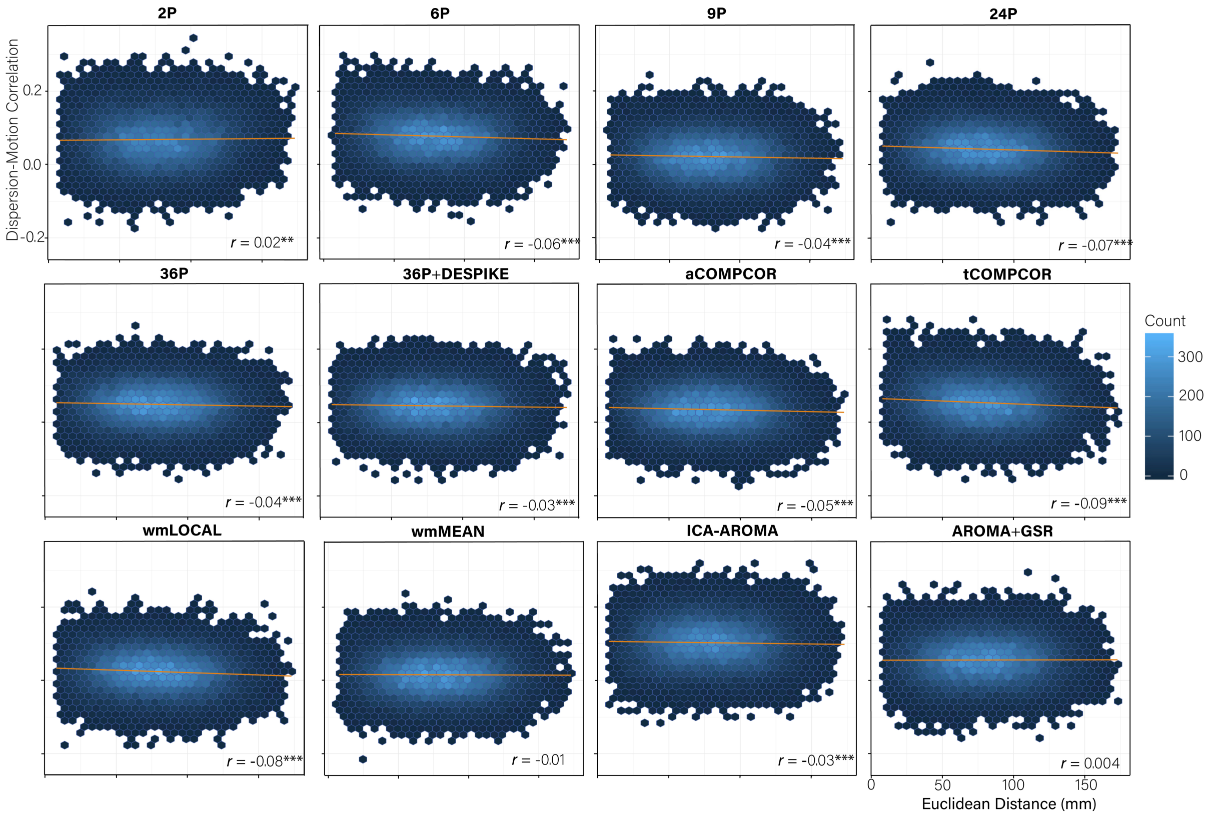

Figure S6. Hexbin plots of the association between the Euclidean distance separating nodes (mm) and the magnitude of the *dispersion-motion* correlation between the edge connecting the nodes (y-axis). A trend line for each pipeline is indicated in blue and the magnitude of the correlation is presented in the bottom right of each panel. *Note*: ***p<0.001.

Marked Heterogeneity in Sub-Network Identification Across Pipelines

There were marked differences across de-noising pipelines in the extent to which sub-networks were identifiable, as operationalized by the *network modularity quality (Q)*. Paired sample *t*-tests revealed significant differences across the *Q* values of most pipelines (Table S3). Neither the difference between the *Q* values from the 2P and the 9P pipelines nor between the tCOMPCOR and wmMEAN pipelines were significant. The 24P pipeline was the least effective at allowing the identification of sub-networks, exhibiting a mean *Q* value of 13.17 (see Figure S7). AROMA+GSR emerged as the most effective pipeline, with a mean *Q* value 19.88. The relatively poor performances of the 24P and 6P pipelines and the relatively good performance of the AROMA+GSR pipeline on sub-network identification parallels the performance of these pipelines in the context in which negative edge weights were not retained (Figure 6 in main manuscript).

Table S3.

Paired samples *t*-test comparisons of the mean *Q* across all pipelines.

	2P	6P	9P	24P	36P	36PDESPIKE	aCOMPCOR	tCOMPCOR	wmLOCAL	wmMEAN	ICAAROMA
**2P**	-										
**6P**	33.21	-									
**9P**	0.53	37.22	-								
**24P**	35.11	8.37	40.08	-							
**36P**	8.57	24.36	12.84	34.02	-						
**36PDESPIKE**	7.31	25.43	10.83	35.17	10.33	-					
**aCOMPCOR**	24.20	-2.29	33.63	-7.31	25.52	26.48	-				
**tCOMPCOR**	14.40	13.05	18.63	17.76	9.18	10.41	18.36	-			
**wmLOCAL**	36.24	6.75	42.06	-2.20	30.92	32.04	6.05	16.47	-		
**wmMEAN**	20.03	18.58	27.30	28.82	13.90	15.42	11.23	1.78	39.67	-	
**ICAAROMA**	33.14	46.30	27.01	47.41	29.79	28.74	40.69	33.22	-47.91	-37.79	-
**AROMAGSR**	42.63	61.27	46.54	62.14	46.45	44.89	57.10	48.34	-62.38	-53.94	-5.85

Notes: Elements of matrix represent *t*-values. Degrees of freedom for all *t*-tests = 392. Columns represent reference level (i.e., positive t-values indicate higher mean *Q* values for the pipeline indicated by the column versus the row heading). Shaded rows indicate non-significant differences between pipelines.

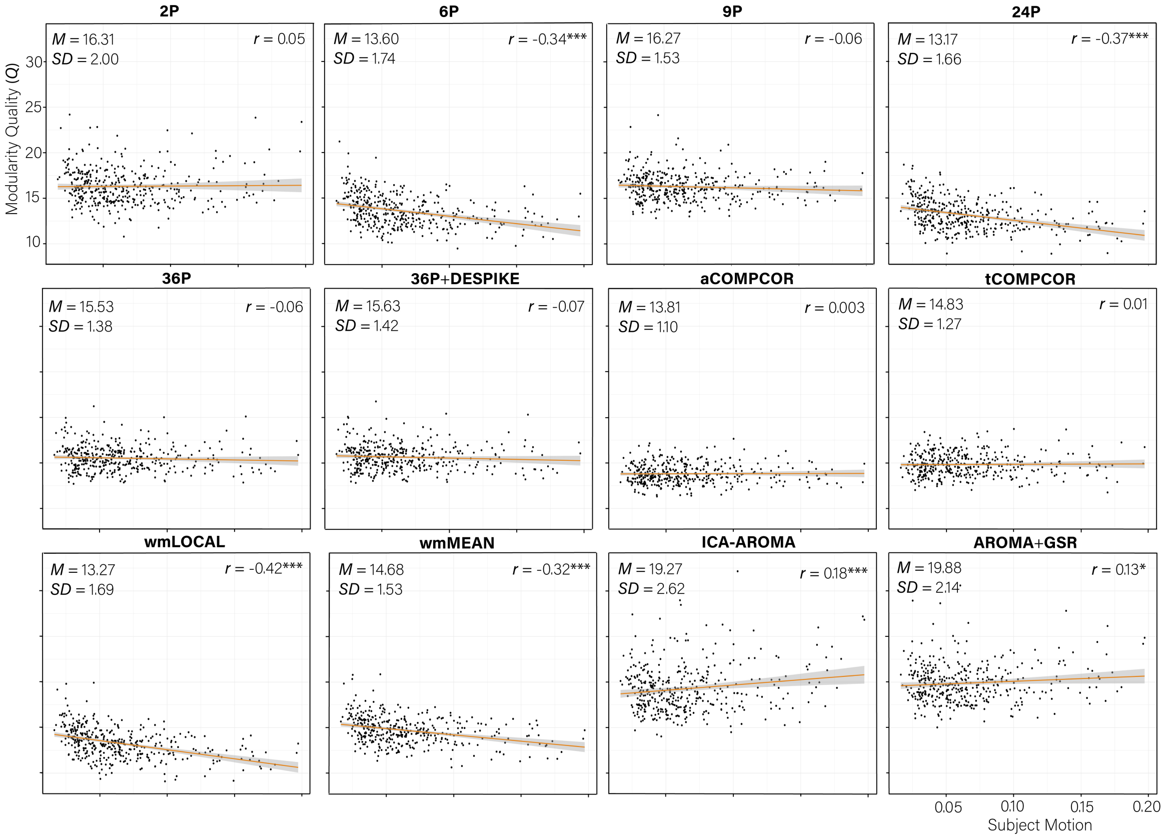

Figure S7. Scatterplot of the association between subject motion (x-axis) and modularity quality (Q, y-axis) with trend line. The partial correlation between subject motion and Q controlling for age and sex is presented in the top right of each panel. The mean Q value for each pipeline is presented in the top left of each panel. ***p<0.001; **p<0.01; *p<0.05.

Q-Motion Associations Reduced by Effective Pipelines

Correlations between *subject motion and Q* ranged between -0.42 and 0.18 (Figure S7). Pipelines that were the least effective at allowing the identification of sub-networks tended to be the pipelines that were the least effective at mitigating subject motion artifacts, $r(10)=0.80$, $p=0.002$. For example, pipelines 6P and 24P performed poorly across both indices while AROMA+GSR and 36P+DESPIKE performed well. Results mirror those in the context in which negative edge weights were not retained (Figure 6 in main manuscript).

Node Flexibility-Motion Correlations Generally Small Across Pipelines

Distributions of *node flexibility-motion* correlations for each pipeline are presented in Figure S8. Paired sample *t*-tests comparing the mean node flexibility-motion correlations cross pipelines are presented in Table S4. Absolute median correlations were small in magnitude across pipelines and ranged between 0.04 and 0.08.

Table S4.

Paired samples *t*-test comparisons of the mean *node flexibility-motion* correlations across all pipelines.

	2P	6P	9P	24P	36P	36PDESPIKE	aCOMPCOR	tCOMPCOR	wmLOCAL	wmMEAN	ICAAROMA
**2P**	-										
**6P**	6.24	-									
**9P**	3.86	2.88	-								
**24P**	2.40	4.44	1.52	-							
**36P**	1.53	4.20	2.27	0.94	-						
**36PDESPIKE**	4.02	1.23	0.87	2.37	5.95	-					
**aCOMPCOR**	7.38	13.90	13.15	12.56	11.14	14.15	-				
**tCOMPCOR**	12.69	18.18	18.40	16.90	16.66	19.92	6.55	-			
**wmLOCAL**	-12.08	8.38	-11.37	-12.51	-11.54	7.81	-21.40	-25.45	-		
**wmMEAN**	9.48	5.14	8.26	9.40	9.70	5.83	-18.76	-24.63	3.94	-	
**ICAAROMA**	1.11	7.24	4.77	3.44	2.35	4.54	6.30	-10.41	12.66	-9.93	-
**AROMAGSR**	4.33	11.03	8.92	7.10	5.68	8.16	4.12	9.24	16.31	13.82	-3.30

Notes: Elements of matrix represent *t*-values. Degrees of freedom for all *t*-tests = 263. Columns represent reference level (i.e., positive *t*-values indicate higher mean flexibility-motion correlations for the pipeline indicated by the column versus the row heading). Shaded rows indicate non-significant differences between pipelines.

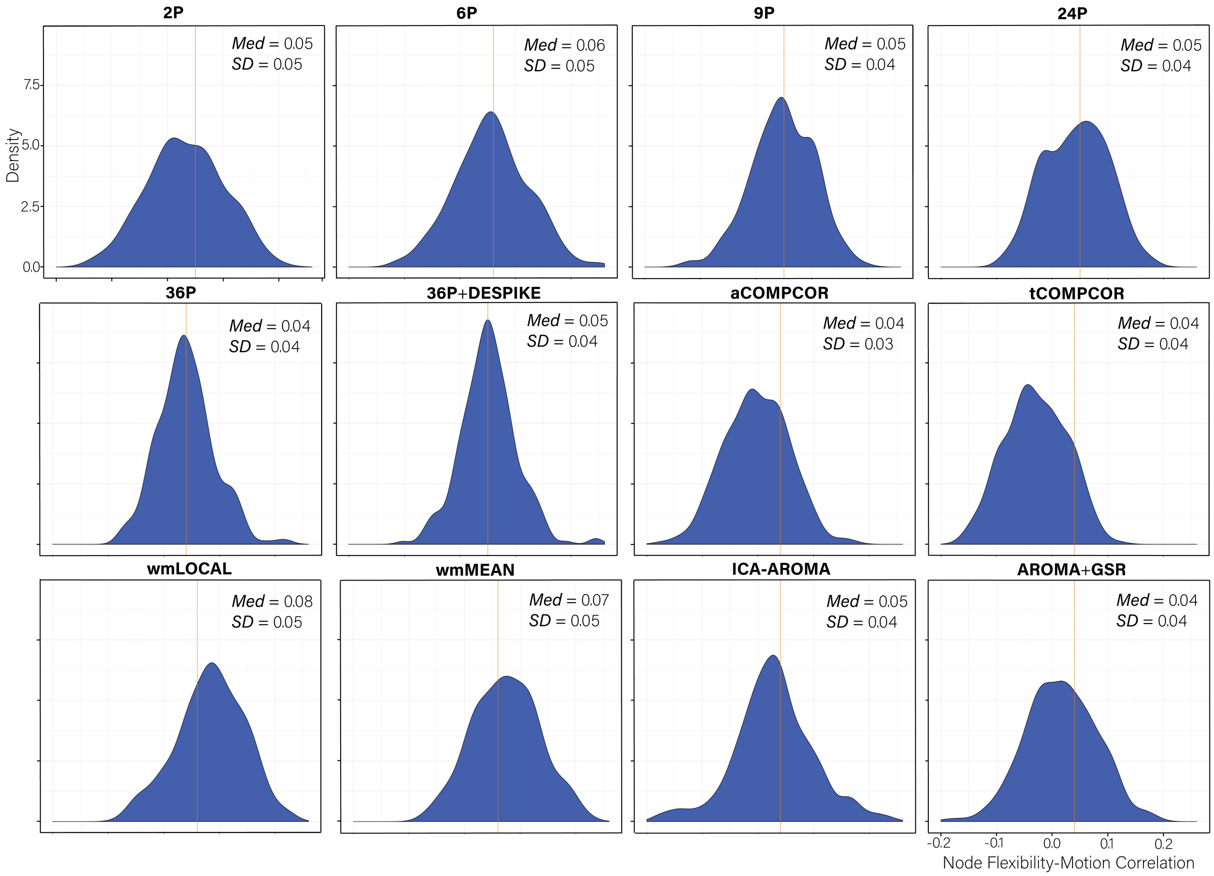

Figure S8. Distributions of all *node flexibility-motion* correlations after de-noising for each pipeline. The median absolute value and the standard deviation of the correlation is displayed in the top right of each panel.

When examining the *percentage of nodes related to subject motion* following de-noising (Figure S9), we found that there was greater variability across pipelines in the extent to which subject motion artifacts were reduced. wmLOCAL and wmMEAN emerged as the least successful pipelines, with over 10% of nodes displaying flexibility values that were significantly associated with motion after de-noising. No nodes displayed flexibility values that were significantly associated with subject motion for the aCOMPCOR pipeline. The locations of the nodes significantly associated with subject

motion are presented in Figure S10. The good performance of aCOMPCOR paralleled its performance in the context in which negative edge weights were not retained (Figure 9 in main manuscript). ICA-AROMA was the most poorly performing pipeline in the context in which negative edge weights were not retained, while in this context it performed better than the wmMEAN and wmLOCAL pipelines.

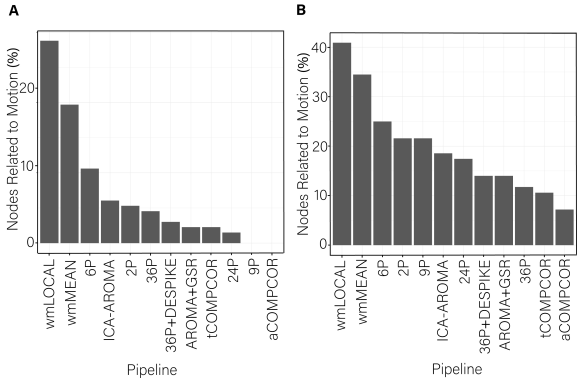

Figure S9. Percent of nodes significantly related to motion after de-noising for node flexibility. More effective de-noising pipelines reduced the relationship between flexibility and motion. Bars are ordered such that the least effective de-noising pipelines are on the left and the most effective are on the right. Panel A illustrates the results after correcting for multiple comparisons. Panel B depicts the results without controlling for multiple comparisons.

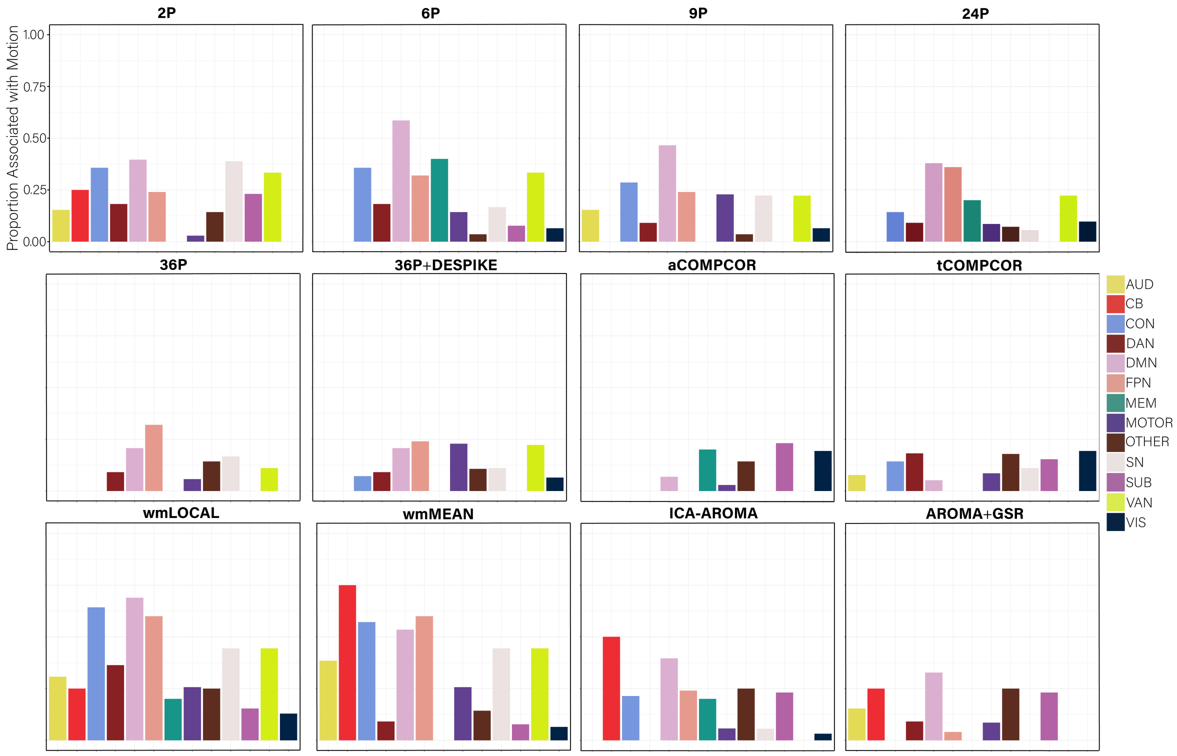

Figure S10. The proportion of nodes within each subnetwork of the Power *et al*. (2011) parcellation with flexibility values significantly related to subject motion, without controlling for multiple comparisons. AUD=auditory; CB=cerebellum; CON=cingulate-opercular network; DAN=dorsal attention network; DMN=default mode network; FPN=frontoparietal network; MEM=memory network; SN=salience; SUB=subcortical; VAN=ventral attention network; VIS=visual network.

When examining the association between *global flexibility and subject motion* (Fig. S11), no significant associations between subject movement and global flexibility emerged for 3 (aCOMPCOR, ICA-AROMA, AROMA+GSR) of the 12 pipelines. wmLOCAL emerged as the least effective pipeline for mitigating the association between subject motion and global flexibility. The good performance of aCOMPCOR was consistent with its performance in the context in which negative edge weights were not retained (Figure 11 in main manuscript).

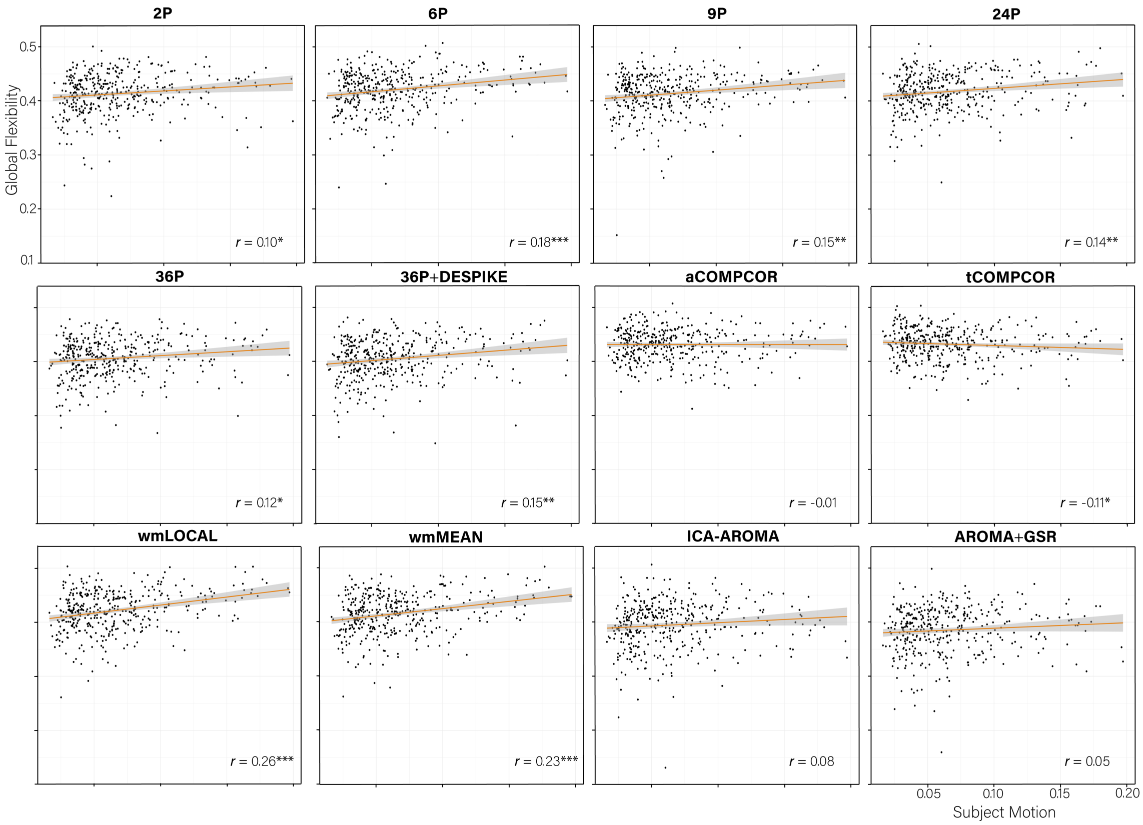

Figure S11. Scatterplot of the association between subject motion (x-axis) and global flexibility (y-axis) with trend line. The partial correlation between subject motion and global flexibility controlling for age and sex is presented in the bottom right of each panel. ****p*<0.001; ***p*<0.01; **p*<0.05.

Node Promiscuity-Motion Correlations Are Generally Small Across Pipelines

Distributions of *node promiscuity-motion* correlations are presented in Figure S12. Paired sample *t*-tests comparing the mean node promiscuity-motion correlations cross pipelines are presented in Table S5. Absolute median correlations were generally small. The values across pipelines ranged between 0.03 and 0.06.

Table S5.

Paired samples *t*-test comparisons of the mean *node promiscuity-motion* correlations across all pipelines.

	2P	6P	9P	24P	36P	36PDESPIKE	aCOMPCOR	tCOMPCOR	wmLOCAL	wmMEAN	ICAAROMA
**2P**	-										
**6P**	21.20	-									
**9P**	-14.63	-43.82	-								
**24P**	9.73	-13.29	25.95	-							
**36P**	-7.99	-27.59	5.79	-21.09	-						
**36PDESPIKE**	-6.57	-25.19	6.56	-18.68	1.98	-					
**aCOMPCOR**	-9.11	-29.73	3.46	-21.32	-2.18	-3.15	-				
**tCOMPCOR**	5.29	-10.97	18.90	-2.36	14.55	13.56	17.02	-			
**wmLOCAL**	16.30	-5.21	39.12	7.76	25.89	23.10	27.40	8.27	-		
**wmMEAN**	0.79	-20.62	18.39	-9.29	10.79	9.09	11.51	-5.05	-22.14	-	
**ICAAROMA**	-0.76	-20.61	12.37	-8.99	5.97	5.02	7.88	-4.69	-15.32	-1.37	-
**AROMAGSR**	-4.10	-25.68	10.68	-13.75	4.03	3.14	5.99	-8.20	-21.40	-4.75	-3.56

Notes: degrees of freedom for all *t*-tests = 263. Columns represent reference level (i.e., positive *t*-values indicate higher mean promiscuity-motion correlations for the pipeline indicated by the column versus the row heading). Shaded rows indicate non-significant differences between pipelines.

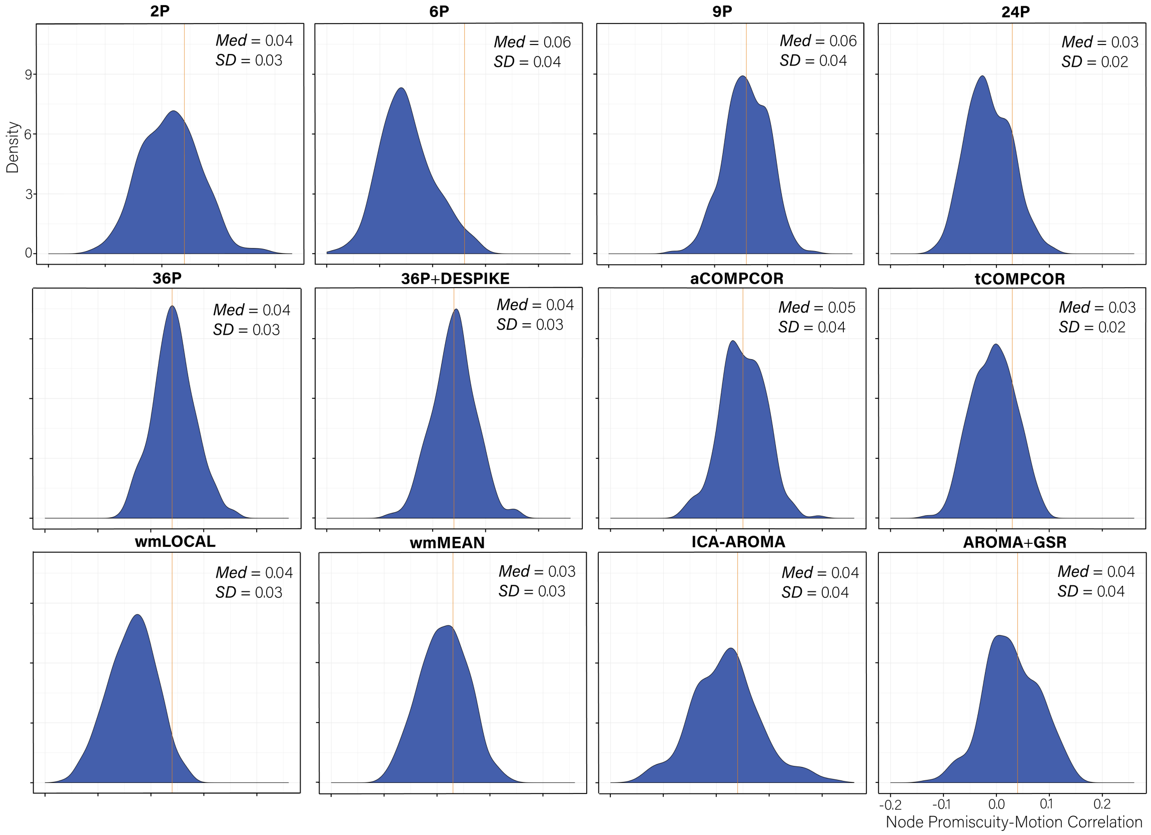

Figure S12. Distributions of all *node promiscuity-motion* correlations after de-noising for each pipeline. The median absolute correlation is displayed in the top right of each panel.

When examining the percentage of nodes significantly associated with subject motion following de-noising, we found that there was marked variability in the effectiveness of reducing motion artifact across pipelines (Figure S13). The ICA-AROMA pipeline had the highest number of nodes significantly associated with motion, although with only 3% of nodes associated with motion after de-noising this performance was still good. The performance of the ICA-AROMA pipeline paralleled its performance in the context in which negative edge weights were not retained (Figure 13 in main manuscript). All other pipelines performed well, with many showing no nodes associated

with motion. There were many more pipelines in the current case with no significant associations with motion than in the case in which negative edge weights were not retained. The locations of the nodes significantly associated with subject motion are presented in Figure S14.

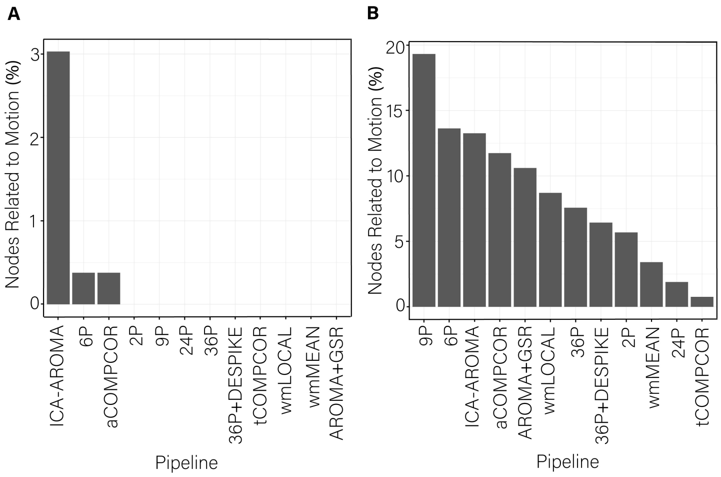

Figure S13. Percent of nodes significantly related to motion after de-noising for node promiscuity. More effective de-noising pipelines reduced the relationship between promiscuity and motion. Bars are ordered such that the least effective de-noising pipelines are on the left and the most effective are on the right. Panel A illustrates the results after correcting for multiple comparisons. Panel B depicts the results without controlling for multiple comparisons.

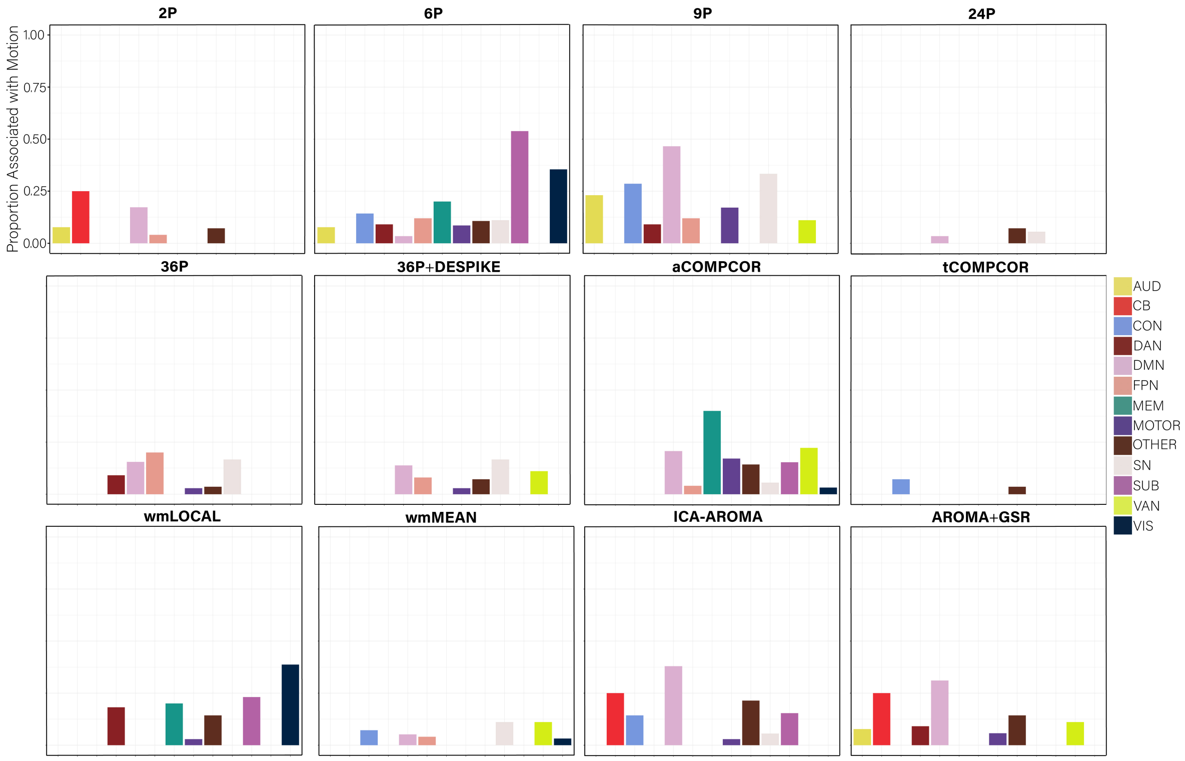

Figure S14. The proportion of nodes within each subnetwork of the Power *et al*. (2011) parcellation with promiscuity values significantly related to subject motion, without controlling for multiple comparisons) AUD=auditory; CB=cerebellum; CON=cingulate-opercular network; DAN=dorsal attention network; DMN=default mode network; FPN=frontoparietal network; MEM=memory network; SN=salience; SUB=subcortical; VAN=ventral attention network; VIS=visual network.

When examining the association between *global promiscuity and subject motion* (Figure S15), no significant associations with motion emerged.

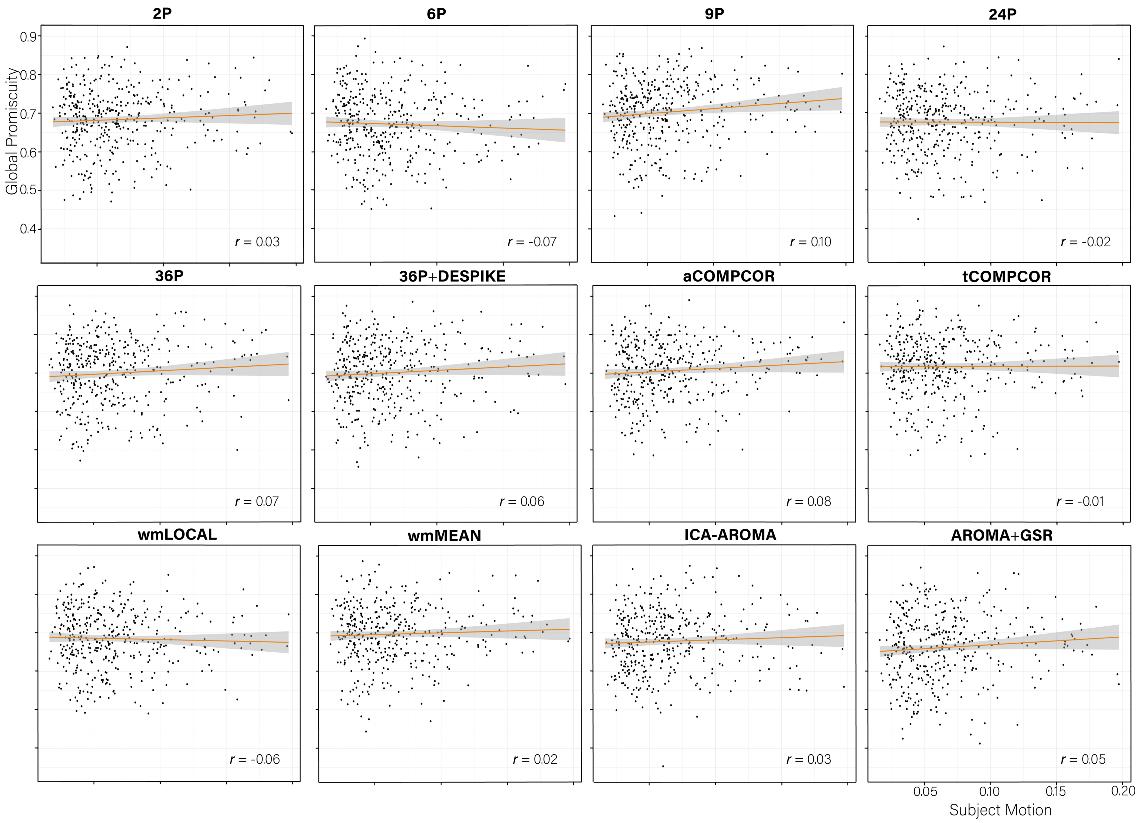

Figure S15. Scatterplot of the association between subject motion (x-axis) and global promiscuity (y-axis) with trend line. The partial correlation between subject motion and global promiscuity controlling for age and sex is presented in the bottom right of each panel.

Table S6.

Paired samples *t*-tests comparisons of the mean dispersion-motion correlations across all pipelines.

	2P	6P	9P	24P	36P	36PDESPIKE	aCOMPCOR	tCOMPCOR	wmLOCAL	wmMEAN	ICAAROMA
**2P**	-										
**6P**	98.54	-									
**9P**	46.58	-51.54	-								
**24P**	123.34	42.36	81.93	-							
**36P**	-12.72	-92.63	-57.83	-136.49	-						
**36PDESPIKE**	-7.49	-87.95	-52.15	-131.02	13.81	-					
**aCOMPCOR**	22.29	-63.68	-19.52	-102.32	37.50	31.97	-				
**tCOMPCOR**	6.53	-85.79	-37.63	-116.86	18.96	13.63	-18.02	-			
**wmLOCAL**	122.89	33.50	79.20	-11.11	123.41	118.02	95.22	111.44	-		
**wmMEAN**	60.34	-34.65	18.31	-72.84	71.77	66.07	36.54	53.38	-90.82	-	
**ICAAROMA**	-51.58	132.53	-90.55	-163.13	-35.29	-40.61	-68.63	-53.66	-159.53	-104.55	-
**AROMAGSR**	10.69	-73.59	-32.53	-106.48	23.25	17.86	-11.98	4.13	-101.07	-46.46	63.11

Notes: degrees of freedom for all *t*-tests = 34715. Columns represent reference level (i.e., positive *t*-values indicate higher mean dispersion-motion correlations for the pipeline indicated by the column versus the row heading). All *p*-values were <0.001.

Table S7

Tests of equality of correlation coefficients representing the association between the Euclidean distance separating nodes (mm) and the magnitude of the *dispersion-motion* correlation between the edges connecting the nodes.

	2P	6P	9P	24P	36P	36PDESPIKE	aCOMPCOR	tCOMPCOR	wmLOCAL	wmMEAN	ICAAROMA
**2P**	-										
**6P**	-24.64	-									
**9P**	17.00	39.96	-								
**24P**	-17.74	4.54	-31.73	-							
**36P**	15.25	34.14	1.38	32.76	-						
**36PDESPIKE**	14.42	33.40	0.47	31.84	-2.09	-					
**aCOMPCOR**	2.01	22.60	-13.16	19.29	-13.85	-13.04	-				
**tCOMPCOR**	-4.04	18.47	-19.56	13.50	-18.66	-17.87	-6.01	-			
**wmLOCAL**	-10.74	14.69	-26.25	9.03	-24.43	-23.54	-12.01	-6.22	-		
**wmMEAN**	-5.22	18.14	-21.94	13.42	-20.57	-19.64	-7.12	-1.22	7.76	-	
**ICAAROMA**	14.67	34.78	-0.76	29.81	-1.96	-1.14	10.99	16.76	23.23	18.24	-
**AROMAGSR**	16.27	35.56	0.46	31.02	-0.88	-0.03	12.41	18.54	24.48	19.86	1.26

Notes: n = 34716. Shaded rows indicate non-significant differences between pipelines. Columns represent reference level (i.e., positive z-values indicate higher mean distance-motion correlations for the pipeline indicated by the column versus the row heading).

Table S8.

Paired samples *t*-test comparisons of the mean *Q* across all pipelines .

	2P	6P	9P	24P	36P	36PDESPIKE	aCOMPCOR	tCOMPCOR	wmLOCAL	wmMEAN	ICAAROMA
**2P**	-										
**6P**	36.42	-									
**9P**	28.74	56.75	-								
**24P**	30.01	-9.69	52.84	-							
**36P**	28.46	56.52	-2.97	53.71	-						
**36PDESPIKE**	29.09	56.96	-4.98	54.15	-9.11	-					
**aCOMPCOR**	-5.10	34.74	38.55	30.79	42.08	43.43	-				
**tCOMPCOR**	-3.79	32.62	32.29	28.61	33.99	34.77	2.00	-			
**wmLOCAL**	29.94	15.09	51.35	-5.57	50.98	51.38	27.90	25.47	-		
**wmMEAN**	-0.04	44.53	35.52	39.12	35.97	36.45	6.14	4.46	46.27	-	
**ICAAROMA**	25.10	48.97	8.74	43.87	9.42	10.11	13.48	13.27	-44.39	-21.26	-
**AROMAGSR**	42.30	66.18	41.60	62.20	33.75	32.05	60.71	50.67	-61.57	-49.76	-24.63

Notes: degrees of freedom for all *t*-tests = 392. Columns represent reference level (i.e., positive *t*-values indicate higher mean *Q* values for the pipeline indicated by the column versus the row heading). Shaded rows indicate non-significant differences between pipelines.

Table S9

Paired samples *t*-test comparisons of the mean *node flexibility-motion* correlations across all pipelines.

	2P	6P	9P	24P	36P	36PDESPIKE	aCOMPCOR	tCOMPCOR	wmLOCAL	wmMEAN	ICAAROMA
**2P**	-										
**6P**	0.81	-									
**9P**	3.11	4.87	-								
**24P**	2.30	1.89	5.86	-							
**36P**	3.80	4.65	1.37	7.33	-						
**36PDESPIKE**	5.18	6.12	3.34	9.06	3.70	-					
**aCOMPCOR**	8.92	8.72	13.35	8.60	15.84	18.95	-				
**tCOMPCOR**	18.13	18.87	24.50	18.90	25.96	29.12	12.61	-			
**wmLOCAL**	4.56	6.48	2.31	8.40	0.70	1.25	-14.72	-25.18	-		
**wmMEAN**	5.35	7.60	3.71	9.54	1.92	0.13	-15.71	-27.16	1.71	-	
**ICAAROMA**	3.31	3.73	0.27	5.11	0.78	2.18	-10.44	-18.12	-1.38	-2.17	-
**AROMAGSR**	0.71	1.38	2.44	2.85	3.33	4.70	9.21	-17.75	-3.92	-4.73	-2.51

Notes: Degrees of freedom for all *t*-tests = 263. Columns represent reference level (i.e., positive *t*-values indicate higher mean flexibility-motion correlations for the pipeline indicated by the column versus the row heading). Shaded rows indicate non-significant differences between pipelines.

Table S10

Paired samples *t*-test comparisons of the mean *node promiscuity-motion* correlations across all pipelines.

	2P	6P	9P	24P	36P	36PDESPIKE	aCOMPCOR	tCOMPCOR	wmLOCAL	wmMEAN	ICAAROMA
**2P**	-										
**6P**	12.68	-									
**9P**	4.09	19.78	-								
**24P**	12.13	0.14	17.28	-							
**36P**	7.50	19.89	4.82	23.04	-						
**36PDESPIKE**	11.29	23.61	9.32	27.68	8.70	-					
**aCOMPCOR**	3.87	14.70	0.34	17.18	4.56	10.21	-				
**tCOMPCOR**	1.16	10.68	2.09	11.57	6.60	11.43	2.81	-			
**wmLOCAL**	1.00	13.14	6.30	13.16	11.03	15.55	5.99	2.56	-		
**wmMEAN**	3.37	18.46	0.50	18.05	5.45	10.15	0.81	2.08	7.67	-	
**ICAAROMA**	8.27	3.84	11.49	3.47	13.17	16.04	9.51	16.43	-5.72	-9.40	-
**AROMAGSR**	7.71	19.20	4.39	18.83	0.22	4.20	2.95	4.34	-8.13	-3.70	-16.43

Notes: degrees of freedom for all *t*-tests = 263. Columns represent reference level (i.e., positive *t*-values indicate higher mean promiscuity-motion correlations for the pipeline indicated by the column versus the row heading). Shaded rows indicate non-significant differences between pipelines.

Mean *Q* Across Pipelines Controlling for Graph Density

To examine whether differences in the average *Q* value across pipelines was driven by differences in network density across pipelines, we used a multilevel model where subjects' *Q* values across the 12 pipelines were the dependent variables and both pipeline (dummy coded with pipelines with no GSR as the reference category), network density, age, and sex as independent variables. Results (Table S11) revealed that pipelines with GSR had significantly higher *Q* values relative to pipelines without GSR, suggesting that differences in mean *Q* values were not simply a product of differences in network density across pipelines.

Table S11

**FIXED EFFECTS**			
	Estimate	*Standard Error*	*p-value*
**Intercept**	0.64***	0.004	<0.001
**GSR Pipelines**	0.01***	0.001	<0.001
**Network Density**	-0.000001***	0.00000001	<0.001
**Age**	0.00003*	0.00001	0.01
**Gender**	0.001	0.001	0.55
**RANDOM EFFECTS**			
	Estimate	*Confidence Interval*	
**Intercept**	0.01	0.009 – 0.01	
**Residual**	0.01	0.009 – 0.01	
**FIT INDICES**			
**AIC**	-24901.55		
**BIC**	-24856.35		

Notes: GSR Pipelines was dummy coded such that the reference category included pipelines without global signal regression. ***p<0.001, *p<0.05. AIC = Akaike information criterion; BIC = Bayesian information criterion.

References

Murphy, K., Birn, R. M., Handwerker, D. A., Jones, T. B., & Bandettini, P. A. (2009). The impact of global signal regression on resting state correlations: are anti-correlated networks introduced? *NeuroImage*, *44*(3), 893-905.
